# Proposal of an alternative way of reporting the results of comparative simulation studies

**DOI:** 10.3389/fpsyg.2025.1549767

**Published:** 2025-03-18

**Authors:** María Paula Fernández-García, Guillermo Vallejo-Seco, Pablo Livácic-Rojas, Francisco Javier Herrero-Díez

**Affiliations:** ^1^Department of Psychology, Faculty of Psychology, University of Oviedo, Oviedo, Asturias, Spain; ^2^Department of Psychology, University of Santiago de Chile, Santiago, Chile

**Keywords:** Monte Carlo simulation studies, *Analysis Plan*, results tables vs. *Traceability Tables*, *Variability Set*, demo example, repeated measures design results, linear mixed model, information criteria

## Abstract

**Introduction:**

Monte Carlo simulation studies allow testing multiple experimental conditions, whose results are often difficult to communicate and visualize to their full extent. Some researchers have proposed alternatives to address this issue, highlighting its relevance. This article develops a new way of observing, analyzing, and presenting the results of simulation experiments and is explained step by step with an example.

**Methods:**

A criterion is proposed to decide which results could be averaged and which results should not be averaged. It is also indicated how to construct *Traceability Tables*. These tables will show the behavior of the different analytical approaches studied under the chosen conditions and their variability under the averaged conditions. A way of observing the influence of the manipulated variables on the performance of the set of analysis approaches studied is also developed, *Variability Set*. Finally, a way of exposing the procedures that have the best performance in a particular condition is suggested.

**Results and discussion:**

This Analysis Plan for reporting the results of simulation studies provides more information than existing alternative procedures, provides valuable information for method researchers, and specifies to applied researchers which statistic they should use in a particular condition. An R Shiny application is provided.

## 1 Introduction

Monte Carlo simulation experimentation is the usual way in which methodological researchers empirically evaluate the properties of statistical estimators of different analytical approaches (Morris et al., 2019; Siepe et al., 2024). The reliability and validity of these results are of vital importance, both for the applied researcher who needs to make an informed choice as to the best statistic to test their hypothesis, and for the method researcher who wants to continue to dig deeper and find better solutions. Both aspects are essential for dealing with the lack of replicability of results (Boulesteix et al., 2020a; Boulesteix et al., 2020b; Kelter, 2024; Lohmann et al., 2022; Luijken et al., 2024; Seibold et al., 2021; Zivich et al., 2023), probably one of the most debated topics in current science, and undoubtedly the topic that brings together the greatest diversity of experts (applied researchers in all sciences, methodologists, philosophers, journalists, etc.).

The interest in good practices in conducting comparative simulation studies (Pawel et al., 2023) is noteworthy, and there are excellent tutorials, guidelines, and standards on how to design, implement, report, present, and check empirical Monte Carlo simulation investigations (Burton et al., 2006; Morris et al., 2019; Pawel et al., 2023; Paxton et al., 2001; Siepe et al., 2024; White et al., 2024). Moreover, seeking to ensure the replicability of the results, and in order not to make mistakes in their design and implementation, several authors have developed programs and applications to perform these investigations flawlessly (Chalmers and Adkins, 2020; Duncan et al., 2024; Kelter, 2024; Kenny and Wolock, 2024). Other researchers have worked on developing applications to organize the presentation of results in tabular and graphical form (Chang et al., 2023; Gasparini et al., 2021; Meyer et al., 2023), meeting the ideal requirements for presentation in scientific journals.

This research focuses on the exploration, analysis, visualization, reporting, and presentation of the results found in the comparative simulation study in relation to the performance of measures used for evaluating different methods (e.g., Type I error rate, Power, Bias, etc.).

Ideally, the results should be reported narratively and also presented in full in different tables and graphs. This is possible when the number of experimental conditions examined is not excessive (e.g., Bell and Rabe, 2020; Blanca et al., 2023; Debelak, 2019). But when the design is complex and the volume of results is high, methodologists look for alternatives to communicate the results in the best possible way because scientific journals have length restrictions for articles (to differing degrees).

To this end, sometimes authors choose to display all results using multiple graphs (e.g., Adams, 2024; Austin et al., 2015; Finch et al., 2018; Haverkamp and Beauducel, 2017; Michiels et al., 2019). Graphs make the article more readable, and they make it possible to identify general trends, but they also make it difficult to identify and interpret the results precisely and accurately (Luijken et al., 2024; Morris et al., 2019; Siepe et al., 2024). In other cases, authors decide to show only the results they consider most salient (e.g., Fingleton et al., 2017; Tibbe and Montoya, 2022; Tofighi, 2020; Wei and Zhan, 2023), which can create the risk and suspicion that perhaps the authors chose to present only the most convenient results in favor of their hypotheses (Nießl et al., 2021). And in other cases, the authors opt to average the results of various experimental conditions. In a review conducted in three highly relevant scientific journals —Psychological Methods, Behavior Research Methods, and Multivariate Behavioral Research—Siepe et al. (2024) identified this behavior in at least 17% of the investigations evaluated (other examples are De and Onghena, 2022; Livacic-Rojas et al., 2020; Michiels et al., 2019; Svetina et al., 2018). Averaged results allow the identification of dominant behaviors; however, they may unintentionally convey the misleading idea that the results extend to all averaged conditions.

When results are reported in these three ways, usually all the original tabulated results are shown in Supplementary material or at a web address, or the authors state their commitment to provide this information to interested readers. However, reading multiple results in multiple tables makes it difficult to identify exceptional results or behaviors, difficult to identify general trends (even more difficult to identify the strength of those trends), and very difficult to identify which statistical procedure has the optimal behavior in each experimental condition examined. Thus, reporting the results of these very voluminous investigations, providing as much information as possible without omitting relevant information, is not an easy task.

Several investigations have addressed this issue and the solutions proposed can be categorized into two groups: one analytical and the other graphical. The former have explored the possibility of summarizing the complete set of results found in the comparative simulation study. To do so, they have sought to express the relationship between the manipulated variables (the supposed cause, e.g., sample size, variable distribution, autocorrelation, etc.) and the observed variables (the effect to be observed, e.g., Type I error rate, Power, Bias, etc.), thus adding complementary information as well. This has been done in four main ways: using regression techniques (Harwell, 1992; Zumbo and Jennings, 2002), using ANOVA (Chipman and Bingham, 2022), using meta-analytical methods (Harwell, 2003; Harwell et al., 1992), and using response surface methodology (Zumbo and Harwell, 1999). The second group have tried to find a way to graphically represent the results, present them in their entirety, and display them better (Rücker and Schwarzer, 2014).

Despite all these efforts, experts in Monte Carlo simulation research state that “There is no one correct way to present results” (see Section 6.2 in Morris et al., 2019) and that “Figures should therefore ideally be combined with quantitative summaries of results, such as tables” (see Siepe et al., 2024, p.17). In the spirit of giving another chance to the possibility of resolving this issue, we have conducted this research, which we now present.

In this research we develop an alternative way to explore and present the results found in the comparative simulation study in relation to the performance execution of the measures (considered appropriate and necessary by the researcher) for evaluating different methods. This approach allows us to obtain relevant information, different and complementary to the information provided by the analytical and graphical solutions previously mentioned, and also to the information provided by the usual way of presenting simulation results (presenting all results, presenting only a set of selected results, or presenting averaged results). Our proposal involves the exploration and visualization of the results in three phases as follows:

*Phase 1*: Presentation and analysis of the global and specific results related to the analytical procedures under study.

*Phase 2*: Exposure and evaluation of the influence of the manipulated conditions on the variability observed in the execution of the set of procedures under study.

The results found in Phase 1 and Phase 2 will be of great interest and useful for the purposes of method researchers. They may be difficult to understand, however, for applied researchers, who are generally not experts in methodology. To address this issue, some researchers have proposed substantive and practical solutions to communicate simulation research results in a user-friendly and straightforward way, and thus provide clear guidelines for applied researchers to make informed decisions (Bandalos and Gagné, 2012; Maxwell and Cole, 1995). Still, the complaints and pleas continue (Boulesteix et al., 2020b). In the spirit of aiming to delve into this issue thoroughly and to bridge the gap between method researchers and applied researchers, Phase 3 is proposed.

*Phase 3*: Evaluation and recommendation for use. Presentation of the *analytical procedure(s) of choice* and representation of the conditions in which they present optimum performance.

Thus, after contextualizing, justifying, and presenting the objective of this research, in this paper we exemplify step by step the procedure we propose, and we do so with a selection of results from the simulation study carried out by Livacic-Rojas et al. (2020). We have structured this paper as follows: Section 2 (Materials and methods) is divided into three sections. In Section 2.1, we describe in detail each of the three phases involved in the analysis and presentation of the results of the comparative simulation studies. In Section 2.2, we briefly present the simulation study carried out by Livacic-Rojas et al. (2020) and explain the motivation for this paper. In Section 2.3, we replicate a subset of experimental conditions performed by Livacic-Rojas et al. (2020), the results of which remained hidden in the averaged results of the first table of their paper. With these results, in Section 3 (Results), we exemplify step-by-step the process and development involved in each of the three phases (Sections 3.1, 3.2, and 3.3), and highlight the added value of each of them. We have developed a Shiny application that allows us to obtain some of the most relevant results of this procedure and to visualize the result as a function of the variables manipulated. Section 3.4 explains how it works. Finally, Section 4 of the paper is devoted to the discussion and conclusion of this procedure.

## 2 Materials and methods

### 2.1 Analysis Plan

The content of the three phases in which the results analysis process will be carried out is described, and a diagram of the process is shown.

*Phase 1*: Presentation and analysis of the global and specific results related to the analytical procedures under study. This involves three activities.

*First activity*: Analysis of the average performance of each analytical procedure (AP) and its variability. Taking as the unit of analysis the performance of each of the analytical approaches tested, we will explore the behavior of each of them in the set of all the experimental conditions examined, and also at each level of each of the variables manipulated in the simulation experiment. The variability statistic we use is the coefficient of variation.

The coefficient of variation $[CV=\left(S/\bar{X}\;\right)\times 100$ ] expresses the standard deviation (S) as a percentage of the arithmetic mean $\left(\bar{X}\right)$ (Kelley, 2007), thus providing a *relative* interpretation of the degree of variability independent of the scale of the variable and being a suitable statistic to compare the variability of the same variable in different samples, or of different variables in the same sample (see a detailed explanation in Ospina and Marmolejo-Ramos, 2019). For this reason, it is possible to use it to observe and compare the relative behavior of each of the APs, each one with respect to the others, and to compare the efficacy of each of them under the different conditions investigated (all conditions, only some conditions, and when the results of some of the conditions examined have been averaged).

The CV has been used in methodological research for different purposes, among others, to calculate sample size by controlling sampling error (Chattopadhyay and Kelley, 2016), for assessing variability of quantitative assays (Reed et al., 2002), for detecting outliers in time series data (Nkechi et al., 2022), for sensitivity analysis in Monte Carlo investigations (Menz et al., 2020), etc. We propose to use the coefficient of variation to observe and to compare the vulnerability or instability of the performance of each AP with respect to the other APs in the set of conditions that have been averaged, and thus obtain even more information from the results found in the simulation study, as shown below.

Both calculations, the averages and the CVs, will be highlighted by symbols that will allow us to display the exceptionality of each of the procedures (each one compared to the others), and the distribution of variability in their behavior at each level of each manipulated variable.

*Second activity*: Analysis of the influence of the manipulated variables on the performance of the set of procedures under study. Considering the set of analytical procedures under evaluation as sample units, the influence of all the variables manipulated in the simulation study on the observed effect of interest (Type I error, Power, Bias, etc.) will be analyzed by means of ANOVA (or by regression). This information is very relevant since it will allow us to observe if the observed performance is dominated by an interaction between variables, if any manipulated variable has no influence on the observed performance, or if only some levels of some variable have differential influence on the observed result.

*Third activity*: Presentation and analysis of the specific results. The results shown in the paper will be conditioned to the number of experimental conditions contained in the simulation study. If the number of experimental conditions examined is not excessive, all results will be shown in tabular form (as well as graphically if possible).

If space is not available, the researcher must decide which results to show. The researcher may decide to show a subset of results, or they may decide to average the results of some experimental conditions. We propose to form a useful and non-arbitrary criterion based on the results found in the two previous activities to decide which results to present (and which not to present in the main text of the paper), and to decide which results can be averaged and which cannot. If averaging is chosen, the usual tables of results will become *Traceability Tables*, showing the performance of each of the analysis approaches studied under the desired experimental conditions, and the variability in their behavior under the particular conditions under which they were averaged. As before, the averages and CVs will be highlighted with symbols that will allow easy identification of the performance of the APs.

*Phase 2*: Presentation and evaluation of the influence of the manipulated conditions on the variability observed in the performance of the set of procedures under evaluation. Presentation of the *Variability Set*.

Taking as a unit of analysis a performance measure of the set of procedures under test, we will explore the variability of the performance of the set of procedures in each of the experimental simulation conditions performed. The *Variability Set* will make it possible to display the influence that the variables manipulated in the simulation study have on the execution of the set of analysis approaches under study.

The exploration, presentation, and writing up of the results found in Phase 1 and Phase 2 is of great interest and usefulness for the purposes of method researchers. But for applied researchers, who are generally not experts in methodology, this information is of little use. In the spirit of bridging the gap between method researchers and applied researchers, Phase 3 is proposed.

*Phase 3*: Evaluation and recommendation of use. Presentation of the *AP(s) of choice*, and representation of the conditions in which they present optimum performance.

Taking into account the information contained in the full results tables, in the specific results tables, or in the *Traceability Tables* (depending on which option has been chosen), the methodologist will determine which would be the *statistical procedure(s) of choice*, i.e., the procedure that performs best under the conditions examined, and he or she will represent how it performs in them. The purpose of Phase 3 is to provide the relevant information to which the applied researchers must pay attention, and thus avoid making decisions on the basis of general criteria that may be incorrect.

**Figure 1:**
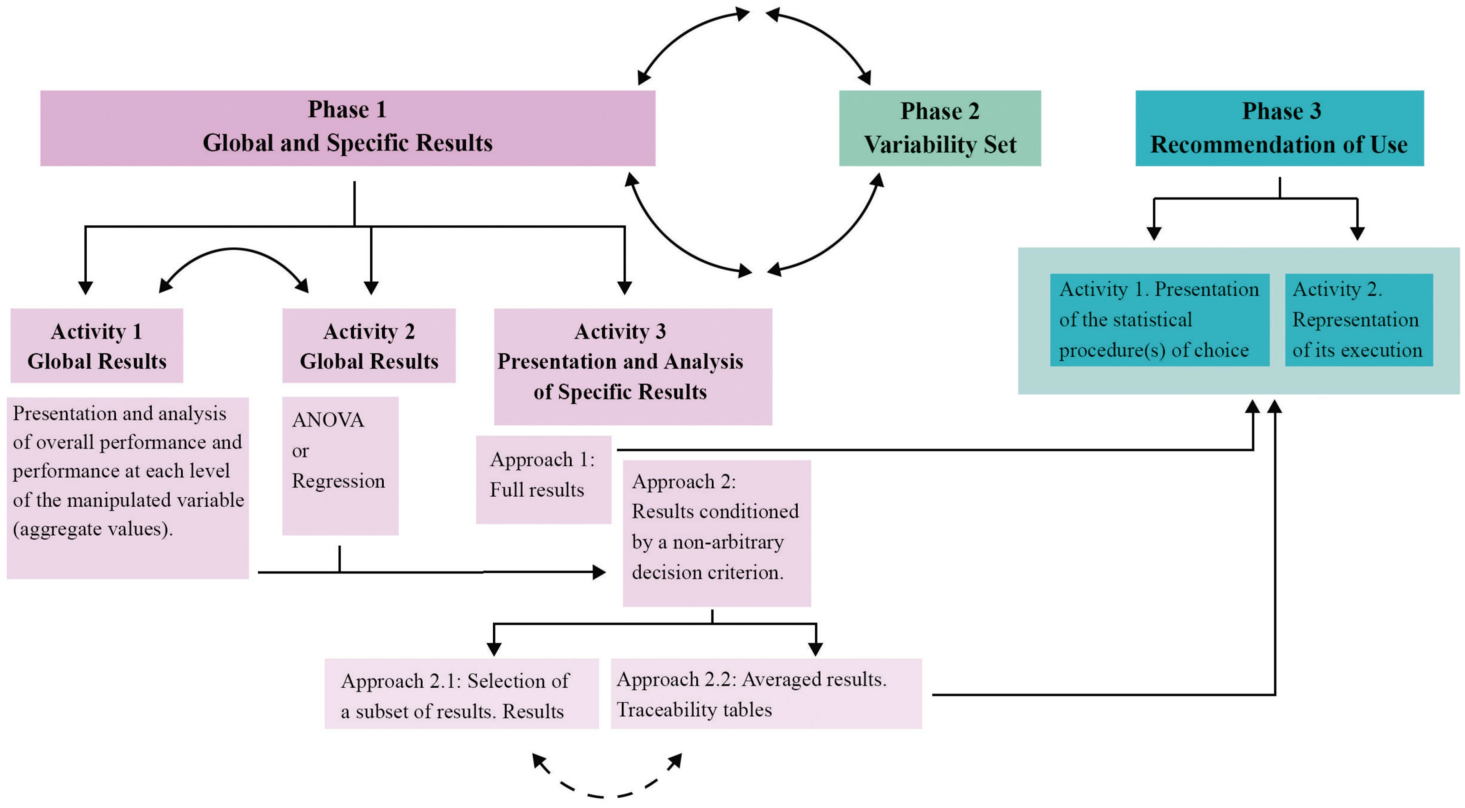
Circle formed by continuous double-arrow arcs = Together, Phase 1 and Phase 2 provide a comprehensive view of the results and integrate everything that has occurred in the simulation research. All of this represents different, distinct, and complementary information; Continuous double-arrow arc = The outcome of both activities, Activity 1 and Activity 2, shapes the objective and non-arbitrary criterion on which the methodologist bases their decision; Discontinuous double-arrow arc = Only one of the two result options is displayed (unless the number of experimental conditions is very large, in which case part of the results might need to be presented in one way, and another part in a different way).

This procedure that we propose will allow method researchers to know the traceability of each of the analytical approaches studied and also to understand how the manipulated conditions determine their influence on the set of analytical approaches studied. It will also allow applied researchers to easily locate the appropriate procedure to use in their particular case, and to understand the risk they are exposed to in making this decision. Overall, we believe that this procedure will contribute to making comparative simulation studies “neutral comparison studies” (Boulesteix et al., 2017; Kelter, 2024) and thereby boost scientific replicability (Boulesteix et al., 2020a; Seibold et al., 2021). To our knowledge, this is the first time that something similar is proposed in order to explore, analyze, display, report, and present the results found in the comparative simulation study in relation to the performance of measures used for evaluating different methods (e.g., Type I error rate, Power, Bias, etc.). Figure 1 schematically illustrates the plan for analyzing the results across the three phases.

### 2.2 Simulation study carried out by, and explanation of the reason for the motivation of this paper

*Contextualization*: When the dependent variable is quantitative, the Linear Mixed Model (LMM) is the best option for analyzing repeated measures designs, as it allows modeling both the fixed effects of the model (i.e., treatment, time, and interaction) and the variance–covariance structure of the data. The combination of the means model and the covariance matrix structure represents the true data-generating process (DGP). If the DGP is correctly identified, the quality of statistical inferences is ensured (see Vallejo et al., 2011). However, the DGP may be partially or entirely unknown.

The LMM enables model selection using the likelihood ratio test (LRT) and/or information criteria (ICs). The LRT is restricted to comparing nested models and can only evaluate two models at a time, requiring a hierarchical approach when more than two models are considered. In contrast, ICs allow for the simultaneous comparison and selection of multiple models, whether nested or not. Due to this flexibility, ICs are widely used and are the focus of extensive methodological research (e.g., Vallejo et al., 2010; Vallejo et al., 2011). One such study is Livacic-Rojas et al. (2020), which examines the behavior of ICs in identifying the DGP across three different scenarios.

Livacic-Rojas et al. (2020) conducted a comparative simulation study to evaluate the performance of five ICs to identify the DGP underlying in a partially repeated measures design (2×5) when the interaction is the term of interest in the model. This was carried out in three scenarios (S). The ICs evaluated were AIC (Akaike IC), AICC (AIC Corrected), HQIC (Hannan-Quinn IC), BIC (Bayesian IC), and CAIC (Consistent AIC) as offered by the SAS PROC MIXED program. In Scenario 1, Means Model, and in Scenario 2, Covariance Structure, the DGP is partially known, whereas in Scenario 3, named Means Model and Covariance Structure, the DGP is completely unknown. The authors manipulated 7 variables, as follows. The data missingness mechanism (MM) [4 levels: Complete Data (CD), Missing Completely at Random (MCAR), Missing at Random (MAR), and Missing Not at Random (MNAR)], the total sample size (*N*) [3 levels: *N* = 50, 100, and 120], the ratio between the size of the groups (R:n_j_) [5 levels: equal size (1), and 4 levels of different size], the homogeneity between the covariance matrices of the groups [3 levels: 1:1, 1:3, and 1:5], the pairing of covariance matrices and group size [3 levels: null, positive, and negative relationship], and the distribution of the measurement variable [5 levels: normal distribution and 4 levels of non-normal distribution]. They also manipulated the covariance matrix (CM) underlying the data. In the first scenario (S1), only the linear random coefficients matrix (RCL) was used, and in the two remaining scenarios (S2 and S3), three CM [heterogeneous first-order autoregressive ARH(1), heterogeneous Toeplitz covariance pattern (TOEPH), and unstructured (UN)] were used.

The combination of the levels of the manipulated variables formed 4,500 experimental conditions whose results would occupy a very large number of tables. It is not possible to present the full set of results in any scientific article due to its length. Thus, the authors chose to average the results. Finally, they presented them in 6 tables, and each of the data points contained therein is the mean of 15 experimental conditions. The average was performed with the set of experimental conditions defined by the interaction of the levels of the manipulated variables [NxR:n_j_].

The results derived from this research are valid and very important, but the content hidden in the average may also be important. In addition, it is impossible to know whether the exposed results can be generalized to all or some of the 15 experimental conditions that have been averaged.

The motivation for developing this alternative approach to presenting the results of comparative simulation studies originates from this investigation and many others like it, where numerous experimental conditions are manipulated and the authors decided to use averages to present their results. Initially, the intention was to explore a way to estimate how much information is lost when the results are averaged. However, the proposed method will allow us to obtain all the information that was highlighted in paragraph 2.1.

### 2.3 Starting point and initial premise

*Starting point*: As a starting point for the explanation of the procedure we propose, we will focus on the first table of results shown by Livacic-Rojas et al. (2020), which has been adapted and is shown in Table 1.

**Table 1:** Results shown in Livacic-Rojas et al. (2020). Average of percentage of occasions on which the ICs identify the true DGP in three Scenarios. Normal distribution and homogeneous covariance matrices.

Scenario 1. Means model					
CM	MM	CD	MCAR	MAR	MNAR
	IC/M%IC_U_ in c’	c’ = [Nx(R:n_j_)] = 15			
RCL	AIC	**95.1**	**95.3**	**95.4**	**95.6**
	AICC	**95.1**	**95.3**	**95.4**	**95.6**
	HQIC	**94.4**	**95.2**	**95.3**	**95.5**
	BIC	**94.5**	**94.7**	**95.4**	**94.8**
	CAIC	**93.5**	**93.6**	**94.7**	**93.8**

Scenario 2. Covariance structure					
CM	MM	CD	MCAR	MAR	MNAR
	IC/M%IC_U_ in c’	c’ = [Nx(R:n_j_)] = 15			
ARH	AIC	71.5	69.2	69.1	68.5
	AICC	75.9	73.1	73.2	72.1
	HQIC	**95.8**	**91.7**	**91.2**	**91.6**
	BIC	**98.6**	**98.8**	**98.5**	**98.5**
	CAIC	**98.8**	**99.1**	**99.4**	**99.5**
TOEPH	AIC	**80.7**	**80.1**	76.7	76.8
	AICC	**84.1**	**82.1**	78.4	78.5
	HQIC	**81.2**	**83.0**	76.5	77.4
	BIC	**80.4**	64.9	57.2	55.5
	CAIC	68.2	53.6	41.9	41.5
UN	AIC	**89.8**	**82.0**	79.4	79.5
	AICC	**87.3**	78.9	75.5	76.8
	HQIC	65.2	63.3	56.2	57.0
	BIC	44.6	31.8	21.1	21.4
	CAIC	25.6	17.8	8.34	8.83

Scenario 3. Mean model and covariance structure					
CM	MM	CD	MCAR	MAR	MNAR
	IC/M%IC_U_ in c’	c’ = [Nx(R:n_j_)] = 15			
ARH	AIC	76.5	76.1	76.0	76.1
	AICC	78.8	78.5	78.5	78.2
	HQIC	**95.7**	**92.8**	**92.7**	**92.9**
	BIC	**99.0**	**98.9**	**98.8**	**98.7**
	CAIC	**99.7**	**99.3**	**99.5**	**99.6**
TOEPH	AIC	**89.8**	**85.5**	**82.8**	**83.7**
	AICC	**90.7**	**86.1**	**83.1**	**83.8**
	HQIC	**82.7**	**83.8**	77.9	78.8
	BIC	**80.7**	65.4	65.1	58.7
	CAIC	71.6	53.5	40.9	44.8
UN	AIC	**90.3**	**85.5**	**80.4**	**80.8**
	AICC	**87.7**	**82.1**	76.7	77.8
	HQIC	65.3	65.7	57.3	56.1
	BIC	44.7	30.8	21.8	21.6
	CAIC	25.7	18.7	9.19	8.93

[IC, information criteria. AIC, AICC, HQIC, BIC and CAIC]; [DGP, data generating process. The means model and the structure of the data covariance matrix constitute the true DGP and may be partially or completely unknown. In Scenario 1, the DGP is completely known. In Scenario 2, the DGP is partially known, and In Scenario 3, the DGP is completely unknown]; [MM, missingness mechanism (CD, MCAR, MAR, and MNAR). The CD condition is considered a special value of the variable MM]; [CM, covariance matrix (RCL, ARH, TOEPH, and UN)]; [M%IC_U_, Average of percentage of occasions on which each ICs identify the true DGP. c’ = experimental conditions that have been averaged]; [*N* = sample size (*N* = 50, 100, and 120)]. [R:n_j_ = ratios between the size of the two groups. R:n_j_ in *N* = 50 (25–25, 20–30, 30–20, 10–40, 40–10), in *N* = 100 (50–50, 40–60, 60–40, 20–80, 80–20, and in *N* = 120 (60–60, 50–70, 70–50, 30–90, 90–30)]. c’ = [Nx(R:n_j_)] = 15, each of the results in the table is the average of 15 experimental conditions. Therefore, this table represents 420 experimental conditions. The details of each scenario, of each level of the manipulated variables, and of the data generated can be found in Livacic-Rojas et al. (2020). Bold = M%IC_U_ higher than 80%. The data presented in this table are the same as those shown in Table 1 by Livacic-Rojas et al. (2020), and the reproduction has been done with the consent of Psicothema and the paper’s authors. However, the acronyms IC/M%IC_U_ in c’ and c’ = [Nx(R:n_j_)] = 15 in the column headings form part of the nomenclature of the procedure we propose in this paper. The order and arrangement of the results have also been changed.

Table 1 shows the performance of each IC in 28 experimental conditions (in S1 [1 CM x 4 MM = 4c] and in each scenario S2 and S3, [3 CM x 4 MM = 12c]) when the distribution is normal, and the covariance matrices are homogeneous. However, each result is the average of the performance in 15 experimental conditions (c’ = [NxR:n_j_] = 3×5 = 15). Therefore, Table 1 condenses the result of 420 experimental conditions. A description of these results can be found in Livacic-Rojas et al. (2020), and they are also described in detail in the Supplementary material. To interpret the results it should be noted that the authors consider that the behavior of the ICs is adequate when the identification of the true DGP ≥80%.

*The initial premise* from which we start is the following. In order for Table 1 to report as much information as possible about the influence of the manipulated variables on the performance of the ICs, the average should represent the set of conditions that have been averaged. If this is so, a subset of the experimental conditions contained in the average should replicate the results shown in Table 1. In this case, for example, the result at the three manipulated sample size levels should be similar.

To check this issue, we asked the authors for the result of 84 experimental conditions contained in Table 1, which result from manipulating the following variables (in S1 [1 CM x 4 MM x 3 N = 12c] and in each scenario S2 and S3, [3 CM x 4 MM x 3 N = 36c]), and only in the condition R:n_j_ = 1. The result is shown in Table 2. This result should be compared with the results shown in Table 1.

**Table 2:** Results provided by Livacic-Rojas et al. (2020). Percentage of occasions on which the ICs identify the true DGP in 84 experimental conditions (c) contained in Table 1 (in S1 [1 CM x 4 MM x 3 N = 12c] and in each scenario S2 and S3, [3 CM x 4 MM x 3 N = 36c]). Normal distribution, homogeneous covariance matrices, and R:n_j_= 1.

Scenario 1. Means model													
CM	MM	CD			MCAR			MAR			MNAR		
	IC/N	50	100	120	50	100	120	50	100	120	50	100	120
RCL	AIC	**93.20**	**99.10**	**99.70**	**93.10**	**99.30**	**99.30**	**91.80**	**99.30**	**99.80**	**92.50**	**98.80**	**99.70**
	AICC	**92.50**	**99.10**	**99.70**	**92.60**	**99.30**	**99.30**	**91.80**	**99.30**	**99.80**	**92.30**	**98.80**	**99.70**
	HQIC	**92.40**	**99.10**	**99.70**	**92.50**	**99.30**	**99.30**	**91.50**	**99.30**	**99.80**	**91.90**	**98.80**	**99.70**
	BIC	**90.35**	**99.10**	**99.70**	**90.30**	**99.30**	**99.30**	**89.60**	**99.20**	**99.80**	**90.30**	**98.80**	**99.70**
	CAIC	**87.20**	**99.10**	**99.70**	**87.10**	**99.30**	**99.30**	**86.50**	**99.20**	**99.80**	**88.20**	**98.70**	**99.70**

Scenario 2. Covariance structure													
CM	MM	CD			MCAR			MAR			MNAR		
	IC/N	50	100	120	50	100	120	50	100	120	50	100	120
ARH	AIC	68.50	70.60	71.70	68.40	72.50	70.70	68.60	71.10	70.90	66.50	70.40	69.90
	AICC	73.80	74.30	73.60	74.20	75.50	72.90	74.80	74.10	74.10	72.60	72.50	72.50
	HQIC	**88.20**	75.00	**93.20**	**89.10**	**94.80**	**93.20**	**87.40**	**93.60**	**93.50**	**87.50**	**93.80**	**93.30**
	BIC	**98.10**	**99.40**	**99.40**	**97.70**	**99.40**	**99.20**	**97.30**	**99.50**	**99.50**	**97.20**	**99.50**	**99.90**
	CAIC	**99.40**	**99.80**	**99.70**	**99.10**	**99.70**	**99.60**	**98.70**	**99.90**	**99.90**	**98.90**	**99.70**	**100.00**
TOEPH	AIC	78.00	**89.10**	**88.80**	65.20	**86.80**	**89.80**	58.90	**84.70**	**86.20**	57.50	**86.10**	**87.30**
	AICC	79.60	**92.10**	**91.20**	64.70	**89.50**	**91.50**	58.50	**86.80**	**88.00**	58.70	**87.10**	**89.50**
	HQIC	74.20	**98.20**	**98.80**	56.80	**94.50**	**97.40**	43.90	**89.60**	**93.80**	46.00	**89.20**	**94.20**
	BIC	49.70	**93.20**	**97.50**	23.90	79.60	**90.60**	17.30	69.60	**82.50**	17.20	69.50	**83.10**
	CAIC	23.60	**86.50**	**93.90**	8.80	65.60	**81.00**	4.80	48.00	67.40	5.70	48.90	69.50
UN	AIC	74.40	**96.60**	**99.30**	64.10	**92.10**	**98.00**	55.90	**87.50**	**94.20**	56.00	**86.40**	**95.50**
	AICC	66.40	**96.00**	**99.20**	57.80	**90.00**	**97.80**	47.10	**84.40**	**92.90**	48.70	**85.20**	**94.00**
	HQIC	48.00	**83.90**	**92.60**	39.80	72.10	**84.40**	31.20	61.90	76.00	32.30	64.70	76.10
	BIC	20.10	51.20	66.60	10.90	75.30	47.70	7.20	23.50	34.40	8.30	22.80	32.90
	CAIC	6.30	28.10	44.10	3.10	15.80	26.10	1.30	9.80	14.70	2.20	9.00	15.50

Scenario 3. Mean model and Covariance structure													
CM	MM	CD			MCAR			MAR			MNAR		
	IC/N	50	100	120	50	100	120	50	100	120	50	100	120
ARH	AIC	74.60	76.80	78.00	75.30	78.20	77.60	74.30	76.30	76.90	73.50	77.20	76.90
	AICC	78.80	79.20	79.30	79.20	**80.30**	78.80	77.70	78.20	78.70	76.40	78.80	78.40
	HQIC	**89.90**	**95.70**	**94.10**	**89.20**	**94.60**	**94.20**	**89.90**	**95.70**	**93.80**	**89.10**	**95.60**	**93.80**
	BIC	**98.10**	**99.40**	**99.40**	**98.10**	**99.70**	**99.40**	**97.70**	**99.30**	**99.40**	**97.20**	**99.30**	**99.10**
	CAIC	**99.40**	**99.80**	**99.70**	**99.30**	**99.80**	**99.90**	**99.00**	**99.80**	**99.80**	**99.10**	**99.90**	**99.80**
TOEPH	AIC	**83.30**	**93.40**	**93.70**	70.50	**91.60**	**93.10**	64.20	**91.00**	**92.90**	65.10	**93.20**	**95.60**
	AICC	**83.70**	**94.90**	**94.90**	70.80	**93.30**	**94.00**	62.20	**92.20**	**93.80**	63.10	**93.40**	**96.40**
	HQIC	76.60	**98.40**	**98.90**	56.00	**94.10**	**97.90**	46.70	**90.50**	**95.10**	46.80	**93.60**	**97.70**
	BIC	50.60	**93.40**	**97.50**	24.90	**80.40**	**89.60**	16.10	68.30	**82.30**	18.20	76.70	**84.70**
	CAIC	23.70	**86.70**	**93.90**	7.70	64.60	79.10	5.10	49.30	69.40	5.11	55.50	69.00
UN	AIC	75.32	**97.36**	**99.75**	64.76	**92.58**	**98.88**	56.55	**88.12**	**94.78**	56.28	**87.82**	**96.56**
	AICC	67.04	**96.80**	**99.99**	58.39	**91.09**	**98.70**	47.19	**85.17**	**92.99**	49.93	**86.82**	**95.57**
	HQIC	49.76	**84.60**	**92.96**	39.84	72.46	**84.49**	32.07	62.20	76.89	33.59	65.67	76.40
	BIC	20.85	52.18	67.76	11.62	75.71	49.06	8.56	25.20	35.50	9.36	23.72	34.52
	CAIC	7.97	29.21	43.24	3.43	17.00	27.06	3.49	10.98	15.22	3.41	9.90	16.93

R:n_j_ in *N* = 50 (25–25), in *N* = 100 (50–50), and in *N* = 120 (60–60). These results should be compared with those shown in Table 1 and examine if these are reproduced in the averaged results. Bold = execution of each CI higher than 80%. For the rest, see Table 1.

It is quickly observed that in some experimental conditions the averaged results (see Table 1) are substantially underestimated with respect to the non-averaged results (see Table 2). This is noticeable when the CM is TOEPH and UN, and when the MM is MAR and MNAR, to a greater extent in S2 than in S3. Table 2 shows that when *N* = 50, only on two occasions is the result ≥80%, and the performance of all ICs differs significantly from the performance observed at *N* = 100 and *N* = 120. It can be concluded, therefore, that the averaged results cannot be generalized to the three levels of sample size, and the impact of the other levels of the variable R:n_j_ on the result is unknown.

To test how important sample size is, we decided to replicate the 84 experimental conditions contained in Table 2, but with a slight variation. Instead of considering *N* = 50, 100, and 120, this time it was *N* = 60, 90, and 120. This was done for two reasons. Firstly, if the simulation study is replicated correctly, the result in *N* = 120 should be the same (with slight variations due to chance), and it would be possible to know to what extent 5 more or 5 less subjects in both groups has an impact on the performance of the ICs. Secondly, Vallejo et al. (2010) used *N* = 30 and 60, and Vallejo et al. (2011) used *N* = 30, 60, and 120 in some conditions identical to these, and our results should converge with theirs in the manipulated conditions that are identical. The results are shown in Table 3.

**Table 3:** Replication of the same 84 experimental conditions carried out by Livacic-Rojas et al. (2020), whose results are presented in Table 2. In this case, *N* = 60, 90, and 100. Percentage of occasions on which the ICs identify the true DGP. Normal distribution, homogeneous covariance matrices, and R:n_j_=1.

Scenario 1. Means model													
CM	MM	CD			MCAR			MAR			MNAR		
	IC/N	60	90	120	60	90	120	60	90	120	60	90	120
RCL	AIC	**95.22**	**98.56**	**99.60**	**95.70**	**98**	**99.60**	**94.80**	**99.10**	**99.90**	**94.70**	**98.50**	**99.50**
	AICC	**95.22**	**98.56**	**99.60**	**95.70**	**98**	**99.60**	**94.80**	**99.10**	**99.90**	**94.70**	**98.50**	**99.50**
	HQIC	**95.16**	**98.56**	**99.60**	**95.58**	**98**	**99.60**	**94.50**	**99.10**	**99.90**	**94.70**	**98.50**	**99.50**
	BIC	**94.52**	**98.50**	**99.60**	**95**	**97.90**	**99.60**	**93.80**	**99**	**99.90**	**94.40**	**98.40**	**99.50**
	CAIC	**92.88**	**98.42**	**99.60**	**93.50**	**97.80**	**99.60**	**92.30**	**99**	**99.90**	**93.50**	**98.30**	**99.50**

Scenario 2. Covariance structure													
CM	MM	CD			MCAR			MAR			MNAR		
	IC/N	60	90	120	60	90	120	60	90	120	60	90	120
ARH	AIC	69.20	72.50	71.70	67.80	73.10	71.70	69.10	70.80	71.30	69.60	71.30	70.30
	AICC	75.10	74.60	73.60	72.80	75.80	74	74.90	73.70	71.90	74.40	74.10	72.60
	HQIC	**90.70**	**92.70**	**93.20**	**90.20**	**93.90**	**93.30**	**92.30**	**93.40**	**97.70**	**90.60**	**91.40**	**93**
	BIC	**98.90**	**99.10**	**99.40**	**98.30**	**98.90**	**99.30**	**99**	**98.70**	**99.80**	**97.40**	**99**	**99.80**
	CAIC	**100**	**99.90**	**99.70**	**99.70**	**99.80**	**99.80**	**99.80**	**99.90**	**100**	**99.50**	**100**	**100**
TOEPH	AIC	**81.20**	**88.40**	**88.80**	73.50	**85.30**	**86.50**	70.30	**88.40**	**87.30**	70.60	**83.10**	**87.10**
	AICC	**84.50**	**91.20**	**91.20**	76.80	**88.20**	**89.10**	70.70	**89.10**	**89.70**	72	**85.50**	**88.50**
	HQIC	**86**	**96.40**	**98.80**	70.20	**89**	**97.10**	60.60	**89**	**94.30**	61.50	**83.70**	**95.40**
	BIC	66.80	**89.50**	**97.50**	41.50	72	**89**	27.90	69.40	**82.30**	28.30	62.50	**81.60**
	CAIC	43.10	**80.30**	**93.90**	17.50	52	**81.50**	8.70	28.10	67.70	10.40	31.30	68.80
UN	AIC	74	**94.98**	**99.30**	73.20	**90.40**	**97.30**	65.10	**85.60**	**94.20**	66.90	**87**	**94.50**
	AICC	70.40	**94**	**99.20**	67.30	**88.60**	**96.50**	59.70	**82.40**	**93**	59.50	**84.10**	**92.90**
	HQIC	52.60	**81.90**	**92.60**	46.60	68.30	**85.20**	37	60.70	75.40	38.20	59.60	76.60
	BIC	23.10	43	66.60	15.90	27.90	47.30	10.30	17.10	33.50	11.40	17.80	35.90
	CAIC	9.20	21.10	44.10	5	11.60	26.80	2.80	5	15.30	3.40	5.70	15.70

Scenario 3. Mean model and Covariance structure													
CM	MM	CD			MCAR			MAR			MNAR		
	IC/N	60	90	120	60	90	120	60	90	120	60	90	120
ARH	AIC	76.50	77.90	77.80	75.90	76.30	75.90	77.10	77.40	77.40	75.90	75.10	76.10
	AICC	79.90	79.40	79.30	79.80	77.50	76.80	79.90	79.90	79	79.30	77.40	78.80
	HQIC	**92.30**	**93.40**	**94.10**	**90.90**	**92.90**	**94.90**	**91.80**	**93.20**	**95**	**91.20**	**93.30**	**94.70**
	BIC	**99**	**99.10**	**99.40**	**98.50**	**99.10**	**99.50**	**99.10**	**99.30**	**99.50**	**98.20**	**98.90**	**99**
	CAIC	**100**	**99.90**	**99.70**	**99.70**	**99.90**	**99.60**	**99.60**	**99.80**	**99.70**	**99.50**	**99.66**	**99.60**
TOEPH	AIC	**88**	**92.90**	**93.70**	**80.60**	**91.60**	**92.10**	75.40	**88.30**	**92.20**	75	**88.70**	**93.20**
	AICC	**89.50**	**94.20**	**94.90**	**80.60**	**92.50**	**93.60**	75.60	**88.80**	**93.60**	75.60	**89.60**	**94.20**
	HQIC	**88.20**	**96.80**	**98.90**	71.30	**91.80**	**97.30**	61.50	**85.90**	**95.80**	62.90	**86.30**	**95.10**
	BIC	67	**89.60**	**97.50**	38.20	73.10	**90.20**	26.30	62.70	**81.50**	26	60.70	**83.20**
	CAIC	43.10	**80.30**	**93.90**	17.10	55.60	**80**	9.60	40.40	67.20	8.80	39.10	70.70
UN	AIC	**82.10**	**95**	**99.60**	76.50	**90.50**	**97.50**	66.30	**87**	**94.20**	66	**86.10**	**89.80**
	AICC	78	**94.20**	**99.50**	70.40	**89.20**	**97.10**	59.80	**83.80**	**93.20**	59.50	**84.10**	**89.80**
	HQIC	59.10	**81.90**	**92.80**	47.70	70.60	**86.30**	37.70	56.90	74.80	37.50	58.70	69.30
	BIC	25.60	43	66.70	17.30	28.50	48.80	9.50	19.70	33.30	11.10	19	27.40
	CAIC	10.40	21.10	44.20	5.10	10	27.40	3.10	5.60	13.40	3.30	5.50	9.80

The *N* variable was slightly altered in replicating the simulation experiment, which is now (*N* = 60, 90, and 120). R:n_j_ [in *N* = 60 (30–30), in *N* = 90 (45–45), and in *N* = 120 (60–60)]. All results shown in this table should be compared with those shown in Table 2 and observe if the result is replicated at *N* = 120 and look at what extent the result is changed at *N* = 60 and *N* = 90 regarding *N* = 50 and *N* = 100. Livacic-Rojas et al. (2020) provide details of each scenario, each level of the manipulated variables, and the data generated. Bold = execution of each CI higher than 80%. For the rest, see Tables 1, 2.

A detailed description of these results and the corresponding discussion is shown in the Supplementary material. What we are interested in highlighting here is that, indeed, when the sample size is *N* = 60 (n_j_ = 30) the performance of all ICs improves appreciably with respect to *N* = 50 (n_j_ = 25) (significantly, in Table 3 the performance highlighted in bold in S2 and S3 appears 9 times versus only 2 times in Table 2). And when *N* = 90 (n_j_ = 45), the performance of all ICs is only slightly worse than when *N* = 100 (n_j_ = 50). These results converge with the trend found in Vallejo et al. (2010) and Vallejo et al. (2011).

Thus, once it has been confirmed that the impact of the sample size (and presumably also the impact of R:n_j_) is dissolved in the average, we proceed in the following section to explain step by step the procedure we propose, which, among other issues, will allow us not to ignore the impact that the variables that have been averaged have on the calculation of the average.

## 3 Results

In this section we exemplify step by step the process and the development involved in each of the three phases contained in the results *Analysis Plan*, and highlight the added value of each of them. The procedure is explained with the results of the comparative simulation study shown in Table 3.

It should be noted that the researcher has all the results in tabular form. They have all the tables on their desk and are predisposed to write their contents in the best possible way. In this case all the possible results are in Table 3, but there could be multiple tables of results.

### 3.1 Phase 1: presentation and analysis of the global and specific results related to the analytical procedures under study

In this case, the performance of five ICs to identify the true DGP with a partially repeated measures design (2×5) when the interaction is the term of interest in the model. The reference used (control condition) to evaluate the performance of the 5 ICs is the performance observed in the DC condition, as this condition allows for comparing the results based on the uncertainty associated with each experimental condition. Phase 1 involves three activities.

#### 3.1.1 First activity. Overall results. Analysis of the average performance of each IC and its variability

Table 4 shows, on the left, the averages of the percentages of identification of the true DGP of each of the ICs (M%IC_U_) in all the conditions examined (OP), and according to each level of each of the variables manipulated, S, CM, MM, and N. On the right, Table 4 shows the respective coefficients of variation (CV_U_). Both calculations, the M%IC_U_ and the CV_U_ are highlighted by symbols that allow us to visualize the exceptionality of each of the ICs (each one versus the others), and the distribution of variability in their behavior at each level of each manipulated variable (aggregated values).

**Table 4:** Results of the performance (aggregated values) of each of the ICs in the set of all examined experimental conditions (OP), as well as at each level of each manipulated variable (S, CM, MM, and N) in the comparative simulation study. The left and right columns respectively show the mean percentages of identification of the true DGP of each IC (M%IC_U_) and the respective coefficient of variation (CV_U_).

IC	M%IC_U_ of identification of the true DGP				CV_U_			
	OP = 84c’	Scenarios [S1 = 12c’; S2, S3 = 36c’]			OP = 84c’	Scenarios [S1 = 12c’; S2, S3 = 36c’]		
	OP	S1^1^	S2	S3	OP	S1^1^	S2	S3
AIC	**83.77¡**	**97.77**	**79.48¡**	**83.38¡**	12.76	2.10*	12.86	10.64
AICC	**84.20¡**	**97.77**	**80.32¡**	**83.56¡**	12.82	2.10*	12.74	11.45
HQIC	**83.39¡**	**97.73**	**80.80¡**	**81.18¡**	20.74	2.16*	21.89	22.79
BIC	69.65	**97.51**	65.15	64.84	46.92	2.42*	50.95	51.56
CAIC	60.39	**97.03**	54.08	54.48	65.82	3.11*	74.13	73.40

	Covariance matrix [RCL = 12c’; ARH. TOEPH, and UN = 24c’]							
	RCL^1^	ARH	TOEPH	UN	RCL^1^	ARH	TOEP	UN
AIC	**97.77**	73.66	**85.09¡**	**85.55¡**	2.10*	4.44^	8.56	13.45
AICC	**97.77**	76.44	**86.63¡**	**82.76¡**	2.10*	3.60^	8.53	16.63
HQIC	**97.73**	**92.92**	**85.58¡**	64.50	2.16*	1.83*	15.50	27.70
BIC	**97.51**	**99.01**	66.85	29.15	2.42*	0.53*	35.30	56.37
CAIC	**97.03**	**99.78**	49.55	13.53	3.11*	0.16*	57.83	88.04

	Missingness mechanism [21c’]							
	CD	MCAR	MAR	MNAR	CD	MCAR	MAR	MNAR
AIC	**86.53¡**	**84.24¡**	**82.44¡**	**81.86¡**	11.96	12.36	13.58	13.34
AICC	**87.43¡**	**84.76¡**	**82.50¡**	**82.12¡**	11.21	12.03	14.11	13.86
HQIC	**89.32¡**	**84.32¡**	**80.31¡**	*79.60* **¡**	13.70	18.71	24.95	24.44
BIC	79.21	70.28	64.84	64.26	32.87	45.08	55.74	55.96
CAIC	70.23	60.90	55.09	55.33	49.05	64.87	77.55	77.21

	Sample size [28c’]					
	*N* = 60	*N* = 90	*N* = 120	*N* = 60	*N* = 90	*N* = 120
AIC	76.65	**86.14¡**	**88.51¡**	12.19	10.22	11.54
AICC	76.66	**86.65¡**	**89.31¡**	13.88	9.20	10.59
HQIC	72.45	**85.57¡**	**92.15¡**	29.68	15.61	9.08
BIC	57.58	70.76	**80.59¡**	64.98	44.30	30.99
CAIC	48.95	60.18	72.04	89.70	65.49	46.01

OP = 84 = Overall performance in all the conditions investigated, of which there were 84. In S1 [1 CM × 4 MM × 3 N = 12c] and in each scenario S2 and S3, [3 CM × 4 MM × 3 N = 36c]. ^1^ = In S1, only the CM RCL is studied. For this reason, the S1 and RCL columns contain the same results. [In the M%IC_U_ columns the following is highlighted: the average percentages of identification of the true DGP over 80% (*satisfactory*) are highlighted in bold; ¡ = sensitive IC, this percentage of identification of the true DGP is not maintained in every one of the conditions that have been averaged]. [In the CV_U_ columns the following is highlighted: bold = *reliable* and *satisfactory* IC estimate in c’; bold and asterisked = *reliable* and *efficacious* IC estimate in c’; ^= *reliable* but *unsatisfactory* IC estimate in c’; + = *unreliable* but *satisfactory* IC estimate in c’]. For the rest, see Tables 1, 3.

To interpret the results of Table 4, and also the results of the successive tables, we establish the following criteria *a priori*:

*With respect to the performance averages*: We consider that an IC has a *satisfactory* average performance in the identification of the true DGP when it is ≥80% (Livacic-Rojas et al., 2020 used the same criteria). We consider it to be *efficacious* when it is ≥90%. In addition, we arbitrarily consider that the behavior of an IC is *reliable* when M%IC_U_ represents the whole of the results on the basis of which it has been calculated, and we consider this to be the case when the difference between the highest and lowest result is not greater than 0.109. When M%IC_U_ ≥ 80% but this condition does not hold in all the averaged experimental conditions, we say that the average estimate of the IC is *sensitive*. These aspects are highlighted in the table as follows. All performance averages ≥80% are highlighted in bold. Now, if this behavior does not hold for all conditions contained in that estimate, the performance is defined as *sensitive* and is highlighted by also adding the symbol [¡].

*Regarding the coefficients of variation*: There is no cut-off point beyond which a CV is considered to indicate strong or weak variability, except the observer’s judgment. The absolute magnitude of the CV is not of interest in the Analysis Plan. What we are interested in is observing the magnitude of the CV corresponding to each performance average in terms of the distribution, arrangement, and location of those magnitudes. The interest lies in showing the relative distribution of the CV magnitudes across the different analytical approaches and experimental conditions, and in this way, identifying patterns of variability. That is, in Phase 1,we propose to use the CV to observe and to compare the vulnerability or inconsistency, and therefore also the stability and robustness, of the performance of each IC with respect to the other ICs in the set of conditions that have been averaged, and thus provide added important information to the performance behavior of the ICs in the simulation study.

The information contained in the CVs will be conditional on the corresponding performance averages, and this information is also highlighted in Table 4 in four ways. One, the CV is highlighted in bold when the performance average of an IC is *reliable* and *satisfactory*. Two, it is highlighted in bold and asterisked [*], when the average performance of an IC is *reliable* and *efficacious*. Three, it is highlighted by the symbol [^] when the average is *reliable* but *unsatisfactory*, and four, it is highlighted by the symbol [+] when the average is *unreliable* but *satisfactory*.

Table 4 contains 70 different results for M%IC_U_ (note that columns S1 and RCL contain the same results). The same is true for the results referring to the CV_U_. Table 4 allows us to appreciate the following:

A. On 40 occasions (57.14%), an IC shows satisfactory behavior in the set of averaged conditions (M%IC_U_ highlighted in bold). However, on 32 occasions (80% of them), the M%IC_U_ is highlighted with the ¡ sign, which means that we have qualified them as *sensitive*. In other words, on these 32 occasions, the M%IC_U_ does not represent the set of averaged conditions, and therefore, in no case can it serve as a reference for use. That is, the result cannot be generalized to the set of conditions that have been averaged (c’). As can be seen, no IC is satisfactory in all the conditions evaluated (see column OP).
B. The performance of all of the ICs in conditions S1 (CM RCL) and [ARH(1)], is *reliable* (see CVs highlighted in bold, with and without the symbol * and CVs highlighted with the symbol ^). In these conditions, only in these (10 occasions, 14.28%), the maximum difference between the highest and lowest percentages of identification of the true DGP of all the conditions manipulated is ≤0.109. In this case, we consider that the mean percentage is representative of the set of percentages involved in the calculation.

However, in ARH(1), AIC and AICC meet the *reliability condition*, but they have neither a *satisfactory* performance (i.e., its execution is not ≥80%; they are 73.66 and 76.44% respectively) nor an *efficacious* one (i.e., its execution is not ≥90%). Thus, the M%IC_U_ and CV are not highlighted in bold. However, the CV is highlighted with the symbol [^]. Therefore, they have no practical use under this condition.

On the other hand, all of the ICs in the S1 condition (CM RCL), and consistent ICs (BIC, CAIC, and HQIC) in the ARH(1) condition meet the *reliability* condition and estimate with *maximum efficacy* on all occasions, with more and fewer subjects, with and without loss of data. Therefore, the M%IC_U_ are highlighted in bold (without any added symbol), and the respective CV_U_ are highlighted in bold and with the [*] symbol. There are 8 occasions (11.42%).

That is, in conditions S1 (CM RCL) and [ARH(1)], the result M%IC_U_ in all of the ICs is maintained in all the conditions involved in the calculation of the average, which in this case are the levels of variables N and MM, and therefore, it can be said that the behavior of the ICs is insensitive to the variation studied in variables N and MM in those conditions.

C. The CV_U_s allow us to appreciate three clusters of behavior, AIC-AICC, HQIC, and BIC-CAIC (with the dash we wish to indicate that the difference in the performance of these ICs is minimal). In Table 4 it can be easily seen that the consistent ICs (BIC, CAIC, and HQIC), to a greater extent BIC and CAIC, are the most sensitive and vulnerable in all the experimental conditions examined (OP) and in scenarios S2 and S3. Also the ICs are the most sensitive and vulnerable to data loss and sample size. In all conditions where the ICs are vulnerable, the CV is not highlighted in bold, and in addition, its corresponding M%IC_U_ is either not highlighted in bold, or it is highlighted with the symbol ¡ (*sensitive*). Therefore, this information containing the average value is misleading and can lead to errors if it is generalized and used as a valid criterion for all situations.
D. Other *surprising* patterns are also identified in Table 4.

*First*: It is clear that the larger the sample size, the greater the efficacy of the ICs, and the more similar the performance of all of them is to each other.

It is common to find higher test power in any analytical procedure when the sample size is higher. In this case this does not seem to be the case, at least not for all ICs in all conditions (recall what was discussed in point B). Table 4 shows a significant change between *N* = 60 and *N* = 90, however, the change experienced at *N* = 90 with respect to *N* = 120 is very small. Moreover, the magnitude of the change is uneven among the ICs. A clear asymmetry can be seen between the performance of AIC-AICC-HQIC and that of BIC-CAIC. In other words, to improve the performance of an IC, increasing the sample size will not always be a practical solution for all of them in all the conditions studied here. In other words, this generalization cannot be made.

*Second*: The similarity of the CV_U_ in MAR and MNAR is noteworthy. Also noteworthy is the equidistance of the magnitude of the CV_U_s in MCAR with respect to CD and with respect to MAR-MNAR.

It has been shown that when the loss mechanism is MNAR, the impact on the performance of analytic approaches is much larger than when it is MAR. It has also been shown that the consequences are not significant (generally) when the loss mechanism is MCAR (see Fernández-García et al., 2018). That is, the impact of data loss on the performance of ICs seems to follow a different pattern from that observed in other analytical approaches. Moreover, here too, an important asymmetry in the performance of ICs is also apparent, and that is that in the performance of AIC-AICC, the MM seems to have very little impact, certainly much less than in the execution of BIC and CAICC.

*Third*: The similarity of the CV_U_s of S2 and S3 is noteworthy.

*In summary*: The overall results examined in this way, using the criteria established *a priori* with respect to M%IC_U_ and CV, and being highlighted in the way shown in Table 4, have enabled us to extract very valuable information. Some of this information is definitive and firm (that referring to the reliable behavior of the ICs in S1 conditions and in ARH in S2 and S3). This information can certainly be generalized to all levels of the rest of the manipulated variables. Moreover, it predisposes us and warns us about where we have to focus or direct our attention in the interpretation, reading, and explanation of the specific results, and thus we are able to explain the causes of the *surprising* patterns we have observed. But before we do so, it will help us, together with the result of the *second activity*, to form the non-arbitrary *decision criteria* on the basis of which to decide which specific results to show in the paper.

#### 3.1.2 Second activity. Global results. Analysis of the influence of the variables manipulated on the execution of the set of procedures under study

This information can be obtained by regression or by analysis of variance (These are the analytical solutions referred to in the introduction). We recommend performing the ANOVA instead of the regression because it is easier to study the simple effects in the ANOVA, but everyone has a preferred way of proceeding. In any case, we recommend emphasizing the effect size more than *p*, especially of the interactions, and we recommend being careful when interpreting interactions of more than 2 variables that are statistically significant.

Table 5 shows the results of the ANOVA [(2x3x4x3) SxCMxMMxN] (excluding S1 because only one CM is examined). The ANOVA shows that all the variables, except S, explain differences in executing the set of ICs (Set-ICs). However, only the sources of variation referring to MM and the interaction CMxN are interpretable.

**Table 5:** ANOVA results [(2x3x4x3) S x CM x MM x N] (excluding S1 because only one CM is examined).

SV^1^	F	*gl*_1;2_	*p*	η^2^	Comparisons of means^**^
S	0.455	1;347	0.501		
CM^a^	73.24	2;347	0.000	0.297	
N^a^	27.32	2;347	0.000	0.136	
MM^b^	5.69	3;347	0.001	0.047	[CD*- MAR = 11.11; CD*- MNAR = 11.54]
CM x N^c^	6.40	4;347	0.000	0.069	*n*_j_ = 30[ARH*-TOEP = 30.775; ARH*- UN = 47.39; TOEPH*- UN = 16.620]
					*n*_j_ = 45[ARH*- UN = 32.118; TOEPH*- UN = 21.853]
					*n*_j_ = 60[ARH*- UN = 20.283; TOEPH*- UN = 20.453]
					ARH[−--]
					TOEPH[*n*_j_45*- *n*_j_30 = 21.223; *n*_j_60* - *n*_j_30 = 31.81]
					UN[*n*_j_45*- *n*_j_30 = 15.989; *n*_j_60* - *n*_j_30 = 27.980; *n*_j_60* - *n*_j_45 = 11.990]

SV = Sources of Variation; ^1^ = we made the ANOVA through a comparison of models (see Ato and Vallejo, 2015); F = Fisher’s F empirical value; *gl*_1;2=_ degrees of freedom of the numerator and denominator of the F statistic; *p = p-*value ( α=0.05) ; η^2^ = effect magnitude (Cohen, 2013); ^a^ = Source of variation not interpretable because it is part of a statistically significant interaction; ^b^,^c^ = Comparisons of means and simple effects for the SVs MM and (CM x N), resp., are presented in the column of Comparisons of means; **error rate corrected by Bonferroni; *indicates the highest mean of the levels of the variables being compared; --- = no difference in means is statistically significant.

Regarding MM, the comparisons of means highlight differences in the performance of the Set-ICs between CD and MAR, and CD and MNAR.

Regarding the CMxN interaction, the simple effects show that when the *N* = 60 (nj = 30), the Set-ICs behave differently in the three CMs studied. However, when *N* ≥ 90 (nj ≥ 45), the statistically significant differences in performance in the Set-ICs are between ARH and UN and between TOEPH and UN.

On the other hand, when the CM underlying the data is ARH, the efficacy of the Set-ICs is the same in all the Ns investigated; when it is TOEPH, the performance in the Set-ICs is similar when nj ≥ 45, and in these cases, they are different from nj = 30. Finally, when the CM is UN, the estimate in the Set-ICs in the three Ns studied is very different.

These results are very illustrative, but nothing tells us to which IC they apply. Nevertheless, it can be seen that these results converge with the results described in the previous section, and therefore, they also predispose us and warn us about where we have to focus or direct our attention in the interpretation, reading, and explanation of the specific results. Prior to that, these results together with the ones in the previous section will allow us to form the non-arbitrary decision criteria on the basis of which to decide which specific results to show in the paper.

#### 3.1.3 Third activity: presentation and analysis of the specific results

The information provided by the two previous *Activities* is very rich, and it is impossible to display and detect in the tables of results that the researcher has on their desk (Table 3, but there could be many tables). Moreover, separately, neither of them allows us to make an accurate judgment about each IC in each of the experimental conditions studied, with the exception, in this case, of the exceptional results revealed in Table 4.

However, we have been able to verify that the result of the ANOVA converges with the patterns that we have observed in Table 4. Thus, together, both activities will allow method researchers to make an informed and non-arbitrary decision about which results to present in the main text of the article when it is not possible to present all results due to space constraints (Note that all results should be made available to readers in some form, on a web page, in the Supplementary material, or available on request from the authors).

The results shown in Table 3 take up little space and could be presented in full. But let us imagine that there is not room for all of them. We would have two options:

*First*: The researcher could choose to present a subset of results.

In this particular case, the author could present the results of S1, and either S2 or S3. This would be justified because the ANOVA did not detect statistically significant differences between S2 and S3, and because M%IC_U_ and CV_U_ (see Table 4) are very similar for the five ICs in S2 and S3 (see point D in section 3.1.1).

The number of results could be reduced even further. One could present the results of S1, S2 (or S3), but as for the MM variable, include only the results in CD, MCAR and MAR (or MNAR), for the same reason as above. That is, because of the similarity of M%IC_U_ and CV_U_ in MAR-MNAR, and because the performance of the ICs at the studied levels of the MM variable is not moderated by the presence of other variables (i.e., they are not part of an interaction, and mean comparisons support this aspect).

*Second*: The researcher may choose to average some results.

Our criterion is that the results of variables that are part of an interaction should never be averaged, and neither should the results of different scenarios. Thus, we can make the decision to average the results of each IC in the set of MM levels.

The averages and respective CVs should be presented together as shown in Table 6. We call this table the Traceability Table. In this case, the CV will show the variability experienced by the performance of each IC in the set of MM levels. If each detail observed in Table 3 (in the tables of the specific results that the researcher has on their desk; see the description of said results in the Supplementary material) can be observed in the Traceability Table (observing both, M%IC_U_ and CV_U_), the purpose of this resource would be justified and the criterion used for averaging will have been the correct one.

**Table 6:** Traceability of each IC submitted to evaluation in the comparative simulation study. Mean percentages of identification of the true DGP of each IC in [N_(IC)_ | S x CM]:MM, and your respective coefficient of variation.

		M%IC_U_ and CV_U_ in the set of missingness mechanisms (including complete data) [4c’]													
		M%IC_U_							CV_U_						
		S1	S2			S3			S1	S2			S3		
		RC	ARH	TOEPH	UN	ARH	TOEPH	UN	RC	ARH	TOEPH	UN	ARH	TOEPH	UN
*N* = 60	AIC	**95.11**	68.93	73.90	69.80	76.35	79.75	72.72	0.50*	1.10^	6.90	6.39^	0.80^	7.60^	10.90
	AICC	**95.11**	74.30	76.00	64.22	79.73	**80.33¡**	66.92	0.50*	1.40^	8.20	8.55	0.40^	8.20^	13.38
	HQIC	**94.99**	**90.95**	69.58	43.60	**91.55**	70.98	45.50	0.50*	1.00*	16.90	16.89	0.70*	17.30	22.51
	BIC	**94.43**	**98.40**	41.13	15.17	**98.70**	39.38	15.87	0.50*	0.70*	44.40	38.80	0.40*	48.90	46.01
	CAIC	**93.05**	**99.75**	19.93	6.10	**99.70**	19.65	5.47	0.60*	0.20*	79.90	56.60	0.20*	81.80	62.18
*N* = 90	AIC	**98.54**	71.93	**86.30**	**89.50**	76.68	**90.38**	**89.65**	0.50*	1.50^	**3.00**	**4.70**	1.60^	**2.50**	**4.50**
	AICC	**98.54**	74.55	**88.50**	**87.28**	78.55	**91.28**	**87.83**	0.50*	1.20^	**2.70**	**5.90**	1.60^	**2.80**	**5.60**
	HQIC	**98.54**	**92.85**	**89.53**	67.63	**93.20**	**90.20**	67.03	0.50*	1.20*	**5.80**	15.20	0.20*	**5.70**	17.40
	BIC	**98.45**	**98.92**	73.35	26.45	**99.10**	71.53	27.55	0.50*	1.72*	15.70	45.70	0.20*	18.50	40.50
	CAIC	**98.38**	**99.90**	47.92	10.85	**99.80**	53.85	10.55	0.50*	0.10*	50.17	68.60	0.10*	35.60	69.60
*N* = 120	AIC	**99.65**	71.25	**87.43**	**96.33**	76.85	**92.80**	**89.65**	0.20*	0.66^	**1.10**	**2.50**	1.30^	**0.80**	4.51*
	AICC	**99.65**	73.02	**89.63**	**95.40**	78.48	**94.08**	**87.82**	0.20*	0.95^	**1.30**	**3.20**	1.40^	**0.70**	5.60*
	HQIC	**99.65**	**94.30**	**96.40**	**82.45¡**	**94.68**	**96.78**	67.02	0.20*	2.40*	2.00*	9.80	0.40*	**1.70**	17.35
	BIC	**99.65**	**99.58**	**87.60**	45.83	**99.35**	**88.10**	27.55	0.20*	0.30*	**8.40+**	33.00	0.20*	**8.30+**	40.55
	CAIC	**99.65**	**99.88**	77.98	25.48	**99.65**	77.95	10.55	0.20*	0.20*	15.80	53.00	0.10*	15.30	69.5

[N_(IC)_ | S x CM]:MM = In the rows, the variable N is presented, with the five confidence intervals (CIs) nested within each level of N. The columns display the experimental conditions resulting from the crossing of the variables S and CM. The execution of the CIs has been averaged (M%IC_U_) across all levels of the variable MM studied, which in this case are 4 (CD, MCAR, MAR, and NMAR). In this case, the CV_U_ represents the variability in the performance of the ICs in the set of levels contained in the manipulation of the MM; + = *unreliable* but *satisfactory* IC estimate in the set of averaged conditions (this condition did not appear in the results shown in Table 4). For the rest of the symbols, see the footnote of Table 4.

The Traceability Table should replicate the results revealed in Table 4, i.e., the results rated as *reliable*, the 3 clusters of ICs that were observed based on their behavior, and one should find therein the explanation for and delimitation of the *surprising* patterns that were observed. It should also reflect the results found in the ANOVA (difference of measures and simple effects). If that is achieved, averaging is justified, and therefore, it will be justified to replace results tables with *Traceability Tables*. In Section 3.1.1 and 3.1.2, we wrote “… Moreover, it predisposes us, warns us, about where we have to focus or direct our attention in the interpretation, reading, and explanation of the specific results.” This is what we meant.

*Item extra*: The Traceability table shows that BIC and CAIC are the worst performing ICs when the CM is UN and TOEPH. Moreover, they are very sensitive to data loss, i.e., their behavior is “scourged” by the data loss mechanism (higher CV_U_s). However, this scourge, which is due to the MM, benefits from an increase in sample size in TOEPH but not in UN. That is, when the covariance matrix is UN, BIC and CAIC are collapsed; they do not react to an increase in sample size. This aspect cannot be clearly seen in Table 3 (in the results tables that the researcher has on their desk), although it can be detected in the Traceability Table.

Thus, although it may be possible to present all the results in the paper, when the results are averaged on the basis of a manipulated variable that does not interact with other variables and the result is observed together with the CV_U_s, we can find information that is *impossible* to see in the results tables (in this case, in Table 3, or in the tables that the researcher has on their desk), and that is also *impossible* to find in Table 4. At least, the aforementioned detail had gone unnoticed by us.

We recommend identifying the Traceability Table. We have done it this way, [N_(IC)_ | SxCM]:MM. What is contained in the table is shown in square brackets. Rows and columns are separated by [|]. In the rows, the variable N is presented, and the five ICs are nested at each level of N. In the columns, the experimental conditions resulting from crossing the variables S and CM are shown. The performance of the ICs has been averaged (M%IC_U_) over all levels of the variable MM, which in this case are 4 (CD, MCAR, MAR, and NMAR). This is represented by [:].

The corollary of the results derived from the three activities of Phase 1 could be the following: the result of the three previous activities has allowed us to verify that the variance and covariance matrix strongly determines the behavior of the ICs. That is, there are ICs suitable for each CM, at least, when the data of a repeated measures design can be explained with a non-additive model such as this one. In other words, there is an IC for each matrix. This aspect is also supported by the fact that the ICs that perform better in each situation are much more robust to variation in the levels of the variables MM and N, and the ICs that are less appropriate in each condition are much more vulnerable to variation in the levels of the variables MM and N (always, of course, taking into account the context of the variables manipulated in this comparative simulation study).

### 3.2 Phase 2: exposure and evaluation of the influence of the manipulated conditions on the variability observed in the execution of the set of procedures under evaluation—construction of the *Variability Set*

For this, we calculate the CV of the set of five ICs (Set-ICs) in each of the 84 conditions resulting from manipulating the variables in the comparative simulation study, and we construct Table 7. We call the information contained in Table 7 the *Variability Set.*

**Table 7:** Distribution of variability in terms of CV in the estimation of the percentage in identifying the true DGP of the set of ICs (Set-ICs) in each of the 84 conditions investigated. *Variability Set*.

S	Matrix	Distribution of variability or the *Variability Set*											
		CD			MCAR			MAR			MNAR		
		30	45	60	30	45	60	30	45	60	30	45	60
S 1	LRC	1.1*	0.1*	0*	1*	0.1*	0*	1.1*	0.1*	0*	0.6*	0.1*	0*
S 2	ARH	16.1	15.1	15.8	17.1	13.4	15.7	16.3	16	17.1	15.7	15.7	16.8
	TOEPH	24.9	**6.5**	**4.5**	45.9	20.3	**6.4**	58.6	36.2	12.1	57	33.5	11.8
	UN	62.6	49.6	30.3	73	62.6	45.1	80.4	74.2	57.7	78.6	73.7	56.3
S 3	ARH	12.1	11.8	11.9	12.1	12.9	13.4	11.8	11.8	12.3	12.1	13.3	12.6
	TOEPH	26.9	**7**	2.4*	49.6	20.2	**7.2**	60.6	29.1	13.8	61.4	30.7	12
	UN	62.4	49.6	30.3	72.8	63.4	44.3	81	72.9	59.2	79	73	64.3

[Distribution of variability or the *Variability Set* = CVs calculated with the mean percentages of all ICs in each of the 84 conditions investigated. These have also been shown in Tables A, B, and C in the Supplementary material (Set-CV). Bold = all ICs are reliable and at least perform satisfactorily in this condition; Bold and asterisked = all ICs are reliable and effective in this condition]. For the rest, see Table 4.

In the Supplementary material, we present the results of Table 3 segmented by scenarios. These are Tables A, B, and C, which contain the results of S1, S2, and S3, respectively. These tables also contain the rows headed by Set-M%IC and Set-CV. The M%IC values have no substantive interpretation; they are of no use. The important information is what we can extract from the CVs. The presentation of all of the CVs makes up the *Variability Set*.

The distribution of the magnitude of the CVs in the *Variability Set* will allow us to observe how, in the LMM, the combination of the variables MM (considering the complete data a special value of the variable) and N, condition the behavior of the Set-ICs in identifying the true DGP established by the means model and by the covariance matrix that underlies the data in each one of the three scenarios. This information is implicit in the results shown in Table 3 (in all the tables that the researcher has on their desk), and also in the results shown in Table 6 (Traceability Table), but it is impossible to see it. The overall results displayed in Tables 4, 5, similar to how they predispose us to observe the specific results (whether they are all displayed, only some are displayed, or averaged results are displayed in the *Traceability Tables*), also predispose us to observe the information displayed in the *Variability Set* in Table 7. However, they do not allow us to extract the information provided by the *Variability Set*. This additional information, which is not readily apparent, could be captured metrically in this way.

We interpret the CVs in the same way as they were interpreted in Table 4 and in the Traceability Table. The results that can be extracted from the *Variability Set* are as follows:

At first glance, what strikes us most are the CVs that are highlighted in bold. In those conditions, and only in those, all five ICs identify the true DGP at least 80% of the time. Now let us examine this in more detail.

In S1 the efficacy of all ICs is always above 90%. We have seen that the ICs do not behave exactly the same in all experimental conditions of S1, however, the difference between them is so small in each condition, and the influence that the MM and N variables have is so small as well, that at no time is the identification of the true DGP less than 90%. For this reason, we qualify S1 in the *Variability Set* as a *safety zone.*

In S2 and S3, when the CM is TOEPH, there are also conditions where it can be recommended to use all five ICs. We note that in CD condition, when n_j_ ≥ 45 all the ICs have a satisfactory performance (Even in S3 when n_j_ = 60, the efficacy of all the ICs is above 90%, and it is therefore another *safety zone*). The loss of MCAR data penalizes the Set-ICs result, but this satisfactory performance is maintained, although only when n_j_ = 60. The conditions observed in the *Variability Set* where we can recommend all the ICs, because all of them identify the true DGP at least 80% of the time, are called *confidence zones.*

At first glance, another very unique aspect that occurs only when the CM is ARH(1) is also striking.

When the ARH(1) matrix intervenes in the true DGP, only in this condition, the Set-ICs do not experience significant change either as a function of MM or as a function of N, and it is the same in both scenarios. We had already observed in Tables 4, 6 that in this condition no IC experiences significant change either as a function of MM or as a function of N. We are not surprised by this aspect; therefore, because this unique behavior occurs in this condition, and it is clearly seen in the *Variability Set*; we call it the *protected zone*.

What we do find surprising now is the following: When the CM is ARH(1), the magnitude of the CVs in S3 with respect to the magnitude of the CVs in S2 drops in all conditions (MMxN) *to the same extent* (we could say it drops *en bloc*). To explain this issue, we had to examine Table 3, and we observe that the identity of ARH(1) in S3 impacts significantly on the AIC and AICC ICs. AIC and AICC do not have the best behavior ever in ARH(1); however, they improve their performance in S3, getting closer to the performance of the consistent criteria, which in this situation show maximum efficacy. For this reason, the CVs are lower in S3. This observation led us to identify other unique phenomena, such as the following, which surprised us even more:

We noticed that this change in behavior is not experienced by the other three ICs, at least not in a significant way. Moreover, AIC and AICC also experience a better performance in TOEPH and UN in S3; however, it does not have an impact on the CVs as in ARH(1), and that is because in TOEPH and UN, AIC and AICC are the best performing ICs, and the margin of improvement they experience is smaller.

Again, at first glance, another very unique aspect is also striking when we focus on the TOEPH and UN CMs, and this issue is in line with what was described above:

If we look at the TOEPH and UN CMs, we observe that the CVs are much higher in UN (likewise in S2 and S3). The results found in Phase 1 showed that when the CM is TOEPH and UN, the AIC and AICC ICs perform best, and the performance in both CMs is similar. Again we go back to Table 3, and also to the Traceability Table, and in them we find the explanation. What is happening is that the more complex the CM is (UN is more complex than TOEPH), the more the performance of the ICs differs (those with the best performance from those with the worst). And in the cases of the ICs that perform the worst, the more complex the matrix, the more they are affected by data loss and by N. This is the reason for the difference in CVs between the TOEPH and UN matrices.

We believe that we would not have noticed this if we had not constructed the *Variability Set*. Furthermore, we believe that we would not have found an explanation for it if we had not analyzed the results as was done in the three activities of Phase 1.

In the *Variability Set*, we can also identify two other aspects that have already been observed. These are as follows:

*One*: The CVs show that the whole set of ICs is sensitive to data loss. The most notable reaction occurs when the MM is MCAR concerning CD. Between MCAR and MAR-MNAR, the difference is much smaller. This occurs in all three scenarios and in the four covariance matrices underlying the data.

*Two*: In both Scenarios, S2 and S3, in both CMs, TOEPH and UN, the CV shows that the increase in the sample size has a systematic effect on Set-ICs, causing a tendency to homogenize behavior. This can be seen by observing how the CV decreases in each condition as the N increases. However, we know that this issue cannot be generalized to the five ICs, as already discussed in the previous section.

*In summary*: From our point of view, the *Variability Set* provides us with information that is very relevant and different from the information provided by the results found in Phase 1. This information is impossible to obtain by looking at the multiple tables of specific results that the researcher might have on their desk or by looking at the Traceability Table. We believe that multiple graphs could not provide this information either.

Before moving on to Phase 3, we’d like to present a metaphor of something that is shown in Figure 1 when the *Analysis Plan* is plotted. Let us imagine that we are about to walk the Camino de Santiago, specifically, the French Camino de Santiago. We would say that Phase 1 would be the equivalent of doing the Camino on foot or by bicycle, and the *Variability Set* in Phase 2 would be like doing the Camino in a balloon or in a light aircraft. When we outlined the *Analysis Plan,* we wrote that the *Variability Set* will allow us to visualize the streams of influence that the variables manipulated in the simulation study have on the performance of the set of analysis approaches under study. We were referring to these results observed by means of the *Variability Set.*

In the next section, we will only provide the information that applied researchers need to make a decision. We will spare the applied researchers, if they wish, this long route, which from our point of view is of great interest, but only the method researchers will appreciate its magnitude.

### 3.3 Phase 3. Evaluation and recommendation of use. Presentation of *analytical procedure(s) of choice* and representation of the conditions in which they present optimum performance

Now the researcher will determine which are the *analytical procedures of choice* (in this case, the *ICs of Choice*), and he or she will present the schematization of their performance. The *ICs of Choice* are easily chosen by looking at the full results tables (if it has been possible to show the results in the paper) or by looking at the *Traceability Table* results (when it has been possible to form a non-arbitrary decision criterion). If the volume of results is very large, the researcher might consider presenting some results one way and other results another way. This information is shown in Table 8.

**Table 8:** Presentation of *analytical procedure(s) of choice* and representation of the conditions in which they present optimum performance.

S	CM	*ICs of choice*	Performance [MM, N]
S 1	LRC	[AIC, AICC] & [HQIC, BIC, CAIC]	$A\text{≡}\left[MM,N\right]$
S 2	ARH	[CAIC, BIC] & HQIC	$A\text{≡}\left[MM,N\right]$
	TOEPH	[AICC, AIC] & HQIC	$B\left[\begin{array}{c} CD:N\\ R_{MM}:n_{j}\geq 45 \end{array}\right]$
	UN	[AIC, AICC] & HQIC	$B\left[MM:n_{j}\geq 45\right]$
S 3	ARH	Same as scenario 2	Same as scenario 2
	TOEPH		
	UN		

ICs of choice = Procedures with the same behavior are shown in brackets. If one is significantly better than another, it is listed first, and so on; Performance [MM,SS] = The brackets indicate the variables used to express the performance of the ICs of choice in each of the conditions defined in the rows (SxCM). Additional brackets may appear if there are more manipulated variables.

The *ICs of Choice* are the ICs that have demonstrated, at least, a satisfactory performance (≥80% of identification of the true DGP) in the set of conditions (or in a subset of conditions) defined by the manipulated variables, in this case, MM and N in each condition defined by the true DGP. It could be the case that no IC was effective.

In the description of the performance of the *ICs of Choice*, first appears the qualification reached according to the empirical efficacy demonstrated. It is highlighted by means of the letters A, B, and C. A indicates that all the *ICs of Choice* achieve an efficacy greater than 90% under the conditions specified (indicated in square brackets). B indicates that the *ICs of Choice* only achieve an efficacy greater than 90% under some of the specified conditions. C indicates that in no case do the *ICs of Choice* reach an efficacy of 90%, but in all or some of the manipulated conditions (the conditions specified in square brackets), the performance is satisfactory; that is, the percentage of identification is ≥80%.

Regarding performance, ≡ indicates that the performance is the same in all conditions expressed in brackets [in this case for example, it is in MM and N (in S1), and then, in S2 and S3 (in CM ARH)]. If the performance is not the same, the conditions under which the ICs have an optimum performance are indicated. For example, the row corresponding to the TOEPH covariance matrix shown in brackets [CD: N, and R_MM_:n_j_ ≥ 45]. This indicates that in the condition of Complete Data, the rating B occurs in all sample sizes, and in the rest of the MMs (R_MM_), only when n_j_ ≥ 45.

This is the information that applied researchers, if they so desire, need to locate to make an informed decision.

### 3.4 Shiny applications

A proof-of-concept Shiny application that can be run locally and demonstrates the main functionalities proposed in this study.

We have developed three Shiny applications in R, designed to facilitate the flexible evaluation and interpretation of the results obtained. These applications allow the use of both the data presented in this article and data from another research paper. The algorithms implemented in R (RStudio release 2024.04.2: Build 764), along with the databases and supplementary documentation (pdf format), are available at the following address (see index):

https://drive.google.com/drive/folders/17WecxmWw2ZsngMGtACN0EYF08jGsOFke?usp=drive_link.

**Figure 2:**
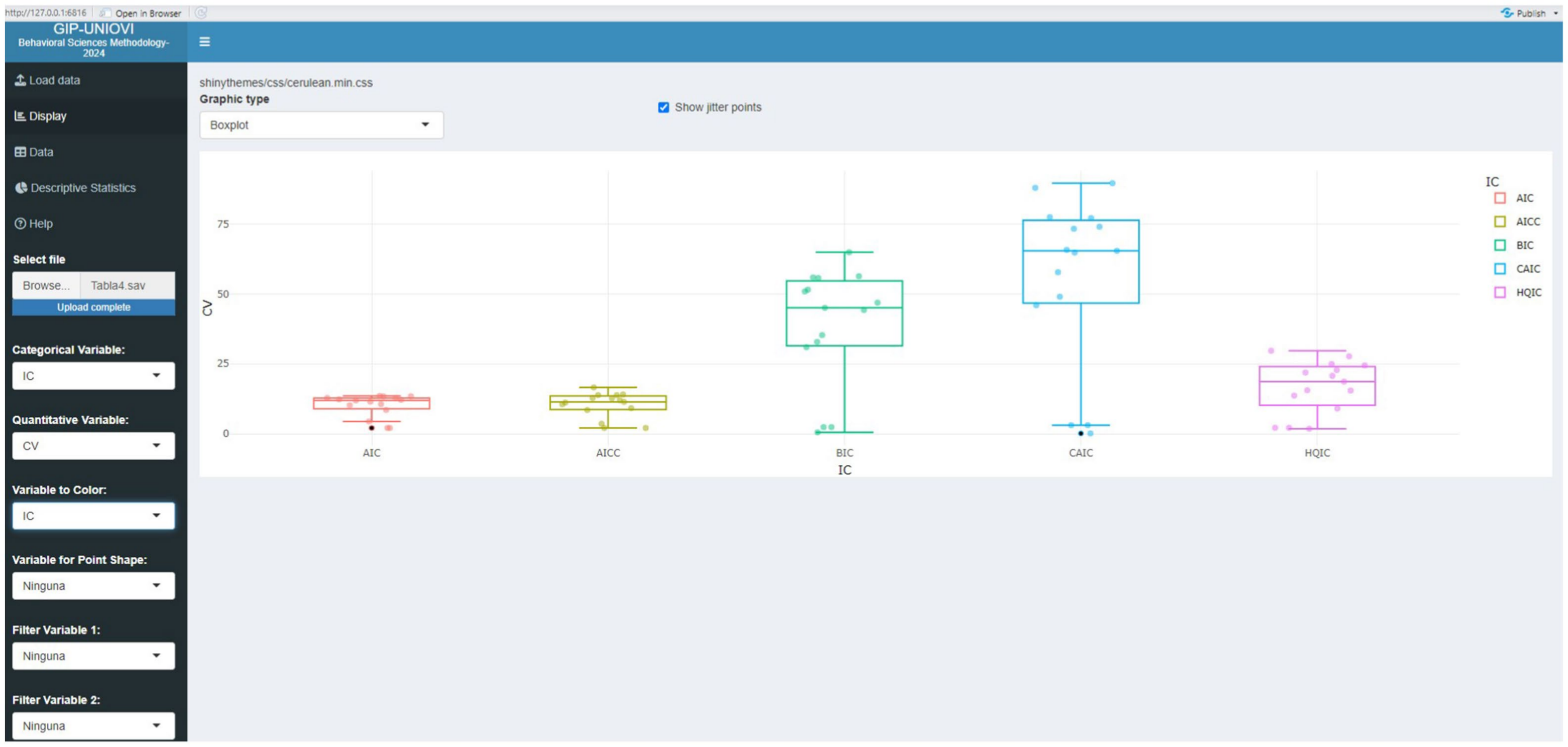
Visualization of selected results from Table 4 using the Shiny application.

**Figure 3:**
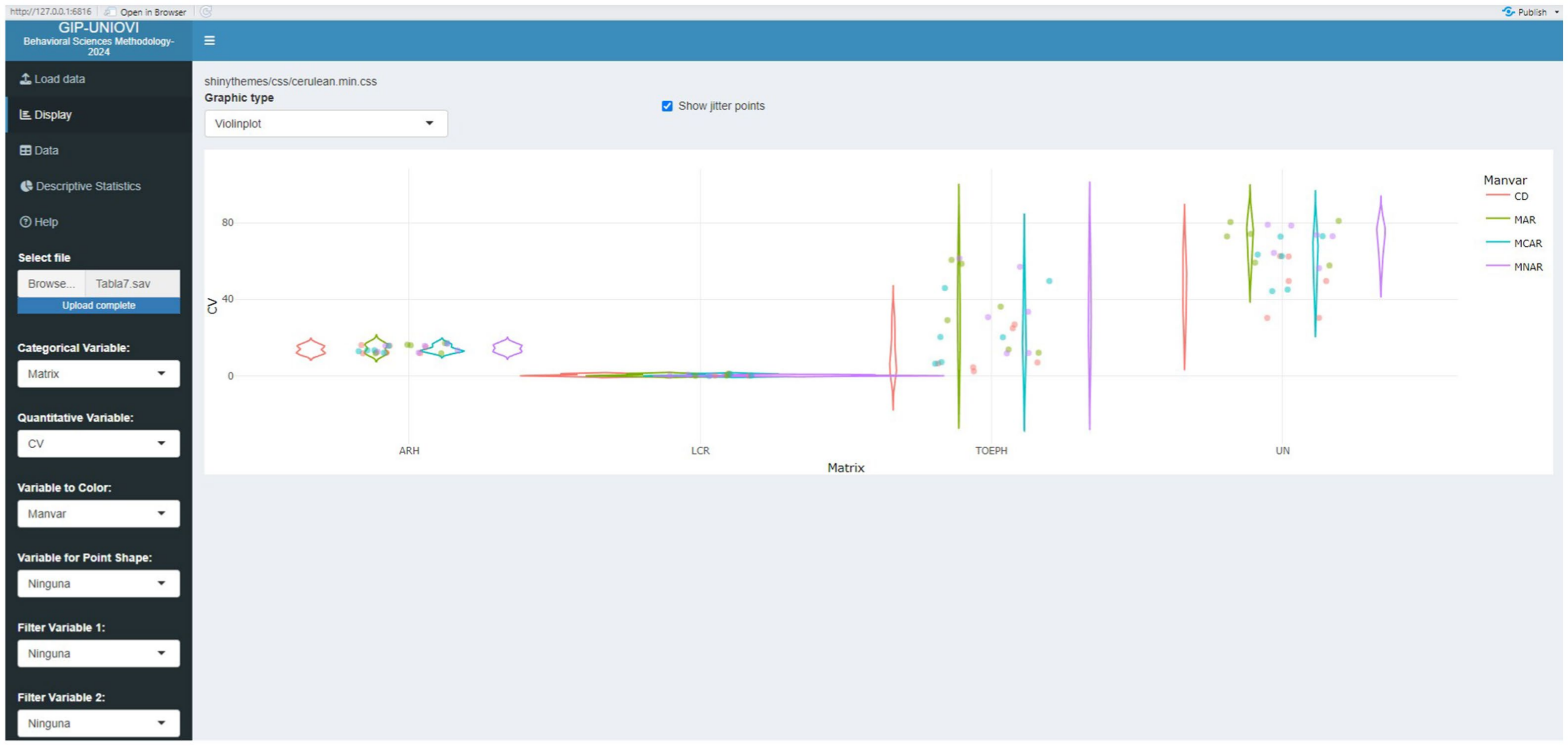
Visualization of selected results from Table 7 using the Shiny application.

Figures 2, 3 graphically represent some results of Tables 4, 7, respectively. This is just an example of how the results presented in the tables can be visualized to aid in their interpretation. These are some of the visualizations that can be obtained using the Shiny application.

## 4 Discussion and conclusions

The main objective of this research was to develop an alternative way of analyzing, presenting, and communicating the results derived from the comparative simulation studies. A three-phase results Analysis Plan is proposed, which is especially useful and necessary when a large number of variables are manipulated in the simulation experiment, which inevitably generates a large number of results.

The contributions and benefits of this Analysis Plan can be summarized in four points:

*First*: In order to report as much information as possible without omitting relevant information, we propose a way of forming a useful and non-arbitrary criterion that allows us to know two things: (1) whether the set of all the results found could be sufficiently represented by a subset of results, and (2) whether some or all of them could be presented in an averaged way without losing the possibility of knowing to what extent and in what way the variables (or levels of variables) on the basis of which the average has been made have an influence.

*Second*: The Analysis Plan makes it possible to extract relevant and novel information that cannot be obtained in other ways of presenting the results. It is the first time that the construction of *Traceability Tables* is proposed when deciding to average some results, and it is the first time that the construction of the *Variability Set* is proposed. The *Traceability Tables* show the performance of each of the analysis approaches studied under the desired experimental conditions, as well as the variability in their behavior under the particular conditions under which they were averaged. The *Variability Set* will make it possible to display the influence that the variables manipulated in the simulation study have on the execution of the set of analysis approaches under study.

*Third*: The examination of the CV provides very valuable information with a different meaning depending on whether it is calculated in the overall average results, in the *Traceability Tables* or in the *Variability Set*. Since the CV must always be accompanied by the corresponding mean values, the symbology proposed for both results allows us to quickly grasp three aspects: the peculiarities of the different analytical approaches under study, the differences between them, and the influence of the manipulated variables both on each analytical approach and on the set of all of them.

*Fourth*: An R Shiny application is provided (still under development) that enables the visualization of some of the most outstanding results of the example presented and that can be used by any researcher who wishes to observe his or her results from this point of view.

The Analysis Plan has been exemplified by the results of the replication of a subset of the experimental conditions of the research conducted by Livacic-Rojas et al. (2020). The purpose of this study was to examine the performance of the AIC, AICC, HQIC, BIC, and CAIC information criteria as provided by the SAS PROC MIXED program to identify the true DGP in a partially repeated measures design when interaction is the term of interest in the model.

Through the example it has been shown that averaging the results without a justified empirical criterion leads to hiding information that may be very relevant, and it also leads to the generalizing of erroneous information to the conditions that have been part of the average. It has also been shown that the proposed Analysis Plan allows us to extract and visualize a great amount of information about the behavior of the CIs, of each one individually, and of the set of CIs as well, including information that was unknown until now. This is because the CV provides a relative measure of variability, making it particularly useful for comparing the stability and performance of different analytical approaches across various experimental conditions.

Beyond the specific results obtained in the simulation experiment for each of the analytical approaches studied, methodologists will be able to identify through the *Variability Set* the safe *zones* and the most vulnerable *zones*, the latter being where the need to advance the research is more imperative due to the higher risk (and potential uncertainty) when choosing the appropriate statistic (such as an IC, in the case of the example shown). *Zones* where no analytical approach is effective may even be identified.

We believe that this Analysis Plan of the results constitutes a novel approach for examining the performance of any analytical approach in the comparative simulation studies, using any relevant measure that the methodologist considers appropriate (Power, Type I error, Bias, etc.). However, we find it necessary to clarify that when the mean of a performance measure is close to zero (e.g., bias), the CV may yield disproportionately high values, which could lead to misleading conclusions if interpreted directly. Therefore, this issue warrants further analysis and should be addressed in future research. Additionally, we have observed that the same CI may perform exceptionally well under certain manipulated conditions while exhibiting poor performance under others. Similarly, within a single experimental condition, substantial variability can be found in the performance of the five CIs under study. This pattern may arise in any analytical method evaluated through simulation studies. In both cases, the CV could also be very high, which is likely attributable to a highly skewed distribution of means. Although the validity of this procedure does not depend on the absolute CV values, in such cases, employing a robust CV instead of the classical CV used here may be warranted (see Ospina and Marmolejo-Ramos, 2019). In other words, it is crucial to determine whether the information provided by a robust CV differs from that offered by the classical CV. Thus, this matter warrants further investigation and should be addressed in future research.

Despite the aforementioned concerns, the example developed demonstrates that the classical CV facilitates a systematic and comparative assessment of variability patterns across multiple conditions, supporting its adequacy for the intended purpose. Therefore, we believe that this Analysis Plan can be applied in all Monte Carlo simulation investigations, regardless of the object of study, and regardless of the set of statistical procedures being studied. For this reason, the Analysis Plan constitutes a new tool that also allows us to reanalyze the results of published simulation experiments to extract additional and complementary information to that already obtained. It will also allow us to map the effectiveness of analytical approaches under multiple experimental conditions, integrating research carried out by different methodologists.

Thus, in line with the previous considerations and the new information obtained during the exemplification of the Analysis Plan, we deem it essential to continue exploring whether relevant new findings are concealed within the remaining averaged results presented in the research by Livacic-Rojas et al. (2020). This issue will be evaluated in two stages. First, we will examine the impact of the unbalanced design on the CIs’ performance and the effect of different relationships between group sizes and covariance sizes. In the second stage, we will apply the Analysis Plan to conditions involving non-normal distributions. Throughout both analyses, we will continue developing the Shiny application to implement a beta version that is accessible online through an appropriate platform.

Finally, we emphasize that Phases 1, 2, and 3 are independent. Therefore, simulation studies may report the results of all three phases (the most comprehensive approach), the first two phases, or only the first phase. In this regard, we recommend that, regardless of whether all findings are reported, only a subset is presented, or averaged results are shown, the *Variability Set* (Phase 2) should also be conducted for two key reasons. First, this is the first time such an analysis has been proposed. Just as in the exemplification of the Analysis Plan, the *Variability Set* allowed us to extract previously unobserved information, this approach may uncover novel and highly relevant insights in other research contexts. Second, beyond its previously highlighted advantages, this analysis could also help identify factors contributing to the lack of result replicability, particularly when certain data analysis approaches are employed.”

Lastly, we argue that applied researchers should have access to essential findings from simulation studies to make well-informed decisions when analyzing their data. This would spare them the challenge of deciphering the complexities of simulation research and the statistical formulations underlying their methods. More importantly, providing clear and structured methodological insights is crucial for fostering rigorous data analysis practices and, ultimately, for ensuring the replicability of scientific findings. To this end, in Phase 3, we propose a structured approach for reporting simulation results in a way that is both accessible and informative. However, our framework is not the only possible solution. What matters is not the specific approach adopted, but rather that methodological findings are effectively communicated to applied researchers. Our Analysis Plan represents one possible strategy to bridge the gap between methodological advancements and their practical application, strengthening both the reliability of statistical analyses and the reproducibility of empirical research.

## Data Availability

The original contributions presented in the study are included in the article/Supplementary material, further inquiries can be directed to the corresponding author. The raw data supporting the conclusions of this article will be made available by the authors, without undue reservation. Additionally, they can be accessed through the Shiny application link.

## Glossary

LMM: Linear mixed model
DGP: Data generating process
IC: Information criteria
AIC: Akaike IC
AICC: AIC corrected
HQIC: Hannan-Quinn IC
BIC: Bayesian IC
CAIC: Consistent AIC
S1, S2 y S3: Scenario 1, Scenario 2 and Scenario 3, respectively
CM: Covariance matrix
CS: Compound symmetry
RCL: Linear random coefficients
ARH (1): Heterogeneous first-order autoregressive
TOEPH: Heterogeneous Toeplitz
*UN*: Unstructured
N: Sample size
MMs: Missingness mechanism
MCAR: Completely randomized
MAR: Randomized
MNAR: Non-randomized
CD: Complete data
CV: Coefficient of variation
