# Supplementary material for: Proposal of an alternative way of reporting the results of comparative simulation studies
Source: Front Psychol. 2025 Mar 18;16:1549767. doi: 10.3389/fpsyg.2025.1549767 (PMC11958989; doi:10.3389/fpsyg.2025.1549767)
Supplement: Supplementary file 1 [file Data_Sheet_1.pdf]

Proposal of an alternative way of reporting the results of comparative simulation studies

Supplementary Material

1 Abbreviations

2. Description of the results presented in Table 1 from the research conducted by Livácic-Rojas et al., (2020), as well as in Table 1 of the main text of this article.

3. Detailed results from the replicated simulation study, which are included in Table 3 of the main text of the article.

Tabla A. Scenario 1. Means model. Percentage of occasions on which the ICs identify the true DGP in [CM RCL x 4 MM x 3 N=12c]. Segregated results from Table 3 of the main text of this article.

Tabla B. Scenario 2. Covariance structure. Percentage of occasions on which the ICs identify the true DGP in [3 CM x 4 MM x 3 SS=36c]. Segregated results from Table 3 of the main text of this article.

Tabla C. Scenario 3. Mean model and covariance structure. Percentage of occasions on which the ICs identify the true DGP in [3 CM x 4 MM x 3 SS=36c]. Segregated results from Table 3 of the main text of this article.

4 Discussion of the results found in the replicated simulation study

5 References

1 Abbreviations in the text of the Supplementary Material. For other abbreviations, see the table footnotes.

DGP: data generating process
IC: information criteria
- AIC: Akaike IC
- AICC: AIC Corrected
- HQIC: Hannan-Quinn IC
- BIC: Bayesian IC
- CAIC: Consistent AIC

S1, S2 y S3: Scenario 1, Scenario 2 and Scenario 3, respectively
CM: covariance matrix
- CS: compound symmetry
- RCL: linear random coefficients
- ARH (1): *heterogeneous first-order autoregressive*
- TOEPH*: heterogeneous Toeplitz*
- *UN: unstructured*

N: sample size
MMs: missingness mechanism
- MCAR: completely randomized
- MAR: randomized
- MNAR: non-randomized
- CD: complete data

CV: coefficient of variation

2 Description of the results presented in Table 1 from the research conducted by Livácic-Rojas et al. (2020), as well as in Table 1 of the main text of this article.

Livácic-Rojas et al., (2020) consider that the performance is satisfactory if the percentage of correct identification is ≥80%.

In S1, all ICs show an identification percentage >90% in all MM conditions. In S2 and S3, when the matrix is ARH, the ICs HQIC, BIC and CAIC show an identification percentage >90% in all MM conditions. AIC and AICC show performance <80%.

The MM does not affect the behavior of the ICs in S1 at all. It also does not affect S2 and S3 when the CM is ARH.

Regarding S2 and S3 when the CM is TOEPH and UN:

In S2, when the CM is TOEPH, the ICs AIC, AICC, HQIC, and BIC perform >80% on CD. AIC, AICC, and HQIQ also perform >80% on MCAR, but BIC experiences a sharp decline in efficacy. When CM is UN, AIC and AICC perform >80% in CD. In MCAR, only AIC. In S2 no CI reaches the level of correct identification of 80% in MAR and NMAR.

In S3, when the CM is TOEPH and UN, AIC and AICC experience slightly better performance than in S2, >80% in all MM in TOEP and only AIC in UN. The rest of the ICs do not experience any change in S3 concerning S2.

In S2 and S3, the execution in MAR and NMAR is the same in all ICs when the CM is TOEPH and UN. In MCAR, the AIC, AICC, and HQIC ICs perform only slightly worse than in CD, and the difference concerning their behavior in MAR-NMAR is very small.

3 Detailed results from the replicated simulation study, which are included in Table 3 of the main text of the article.

1.-In Scenario 1, where the true DGP underlies an RCL matrix, all the ICs identify the true DGP in more than 90% of the cases when n_j =30, with AIC and AICC being the most efficacious and CAIC the least effective (see Table A).

All ICs are sensitive to N. When n_j =45, all ICs identify the true DGP with practically full efficiency, similar to the efficiency performed when n_j =60, and all equally.

The data loss has very little influence on the behavior of the ICs, and only slightly alters their performance in MAR and NMAR (in the same way in both), and only when n_j =30.

In Table A (and also in Tables B and C below), the results contained in the rows headed by Set-M%IC and Set-CV, and the results contained in the columns headed by $M\%IC_U$ and CV_U, belong to the new approach that we propose and will be explained later.

2.-Scenarios 2 and 3 have in common that it is necessary to identify the CM that underlies the data, and in this task, the performance of the ICs is divided. There is a clear difference between the AIC and AICC criteria on the one hand, and BIC and CAIC on the other (see Table B and Table C), and there is also a clear difference in its behavior depending on the CM.

In scenario 2:

Regarding AIC and AICC, they are the most effective when a TOEPH and UN matrix underlies the true DGP, to practically the same extent. If the N is $n_j \geq 45$ they always identify the true DGP in a percentage greater than 80%. If n_j =60, they have a better performance in UN, but if n_j =30, they have the best performance in TOEPH.

BIC and CAIC are the CIs with the worst performance in TOEPH and UN, their behavior being worse in UN than in TOEPH in a notorious way. By the following. In TOEPH, BIC identifies the true DGP in a percentage greater than 80% when n_j =60 in all MM conditions, and when n_j =45 in CD. CAIC identifies the true DGP in a percentage greater than 80% when n_j =60 only on CD and MCAR, and when n_j =45 on CD. However, when the CM is UN, neither identifies the true DGP in a percentage greater than 80% on any occasion.

On the other hand, when the CM is ARH, BIC and CAIC (better the latter) identify the true DGP in a percentage greater than 90%, reaching 100% in some cases, regardless of N and MM.

HQIC has a very particular behavior. When the CM is ARH, it also identifies the true DGP in a percentage greater than 90%, but significantly less than BIC and CAIC. When the CM is TOEPH, if n_j >45, it identifies the true DGP in a percentage greater than AICC and AIC, but if n_j =30 its behavior is always worse. When the CM is TOEP, it performs much better than BIC and CAIC, but notably worse than AIC and AICC, this difference being more notable when n_j =30 and when MM is MCAR, MAR, and NMAR.

In S2 and S3, when the CM is ARH, neither the N nor the MM has an apparent influence on the behavior of the ICs. However, when the CM is TOEH and UN, all procedures without exception are very sensitive to N and MM, much more HQIC, BIC, and CAIC than AIC and AICC. For this reason, there is a notable difference in the behavior of n_j =45 concerning n_j =30. However, the difference between n_j =45 and n_j =60 is very small. As far as the MM is concerned, the performance of all ICs is worse when the data loss mechanism is more aggressive. However, the deterioration in behavior is not linear. The greatest impact on IC behavior occurs between CD and MCAR. Between MCAR and MAR, the deterioration is less, and between MAR and NMAR, the deterioration is even less. Furthermore, this happens more strongly at n_j =30 and n_j =45, but it hardly makes itself felt at n_j =60.

Table A. Scenario 1. Means model. Percentage of occasions on which the ICs identify the true DGP in [CM RCL x 4 MM x 3 N=12c]. Segregated results from Table 3 of the main text of this article.

**MM**	**CD**			**MCAR**			**MAR**			**MNAR**				
**IC/n_j**	**30**	**45**	**60**	**30**	**45**	**60**	**30**	**45**	**60**	**30**	**45**	**60**	**M%IC$_U$**	**CV$_U$**
**AIC**	**95.22**	**98.56**	**99.60**	**95.70**	**98**	**99.60**	**94.80**	**99.10**	**99.90**	**94.70**	**98.50**	**99.50**	97.77	2.10
**AICC**	**95.22**	**98.56**	**99.60**	**95.70**	**98**	**99.60**	**94.80**	**99.10**	**99.90**	**94.70**	**98.50**	**99.50**	97.77	2.10
**HQIC**	**95.16**	**98.56**	**99.60**	**95.58**	**98**	**99.60**	**94.50**	**99.10**	**99.90**	**94.70**	**98.50**	**99.50**	97.73	2.16
**BIC**	**94.52**	**98.50**	**99.60**	**95**	**97.90**	**99.60**	**93.80**	**99**	**99.90**	**94.40**	**98.40**	**99.50**	97.51	2.42
**CAIC**	**92.88**	**98.42**	**99.60**	**93.50**	**97.80**	**99.60**	**92.30**	**99**	**99.90**	**93.50**	**98.30**	**99.50**	97.03	3.11
**Set-M%IC**	94.60	98.52	99.60	95.10	97.94	99.60	94.04	99.06	99.90	94.40	98.44	99.50		
**Set-CV**	1.06	.06	.00	.99	.09	.00	1.12	.06	.00	.55	.09	.00		

Note. The N sample size (N=60, 90, and 120 for n_j= 30, 45, and 60 resp.); Set-M%IC= mean of the percentages identifying the true DGP of the set of ICs in each c represented in the table; Set-CV= coefficient of variation. The value of the standard deviation (SD) is easily estimated using the calculation [(CV/100) x M%IC] o [(CV/100) x M%IC$_U$]. The SD is not shown in the tables for the economy of space. The means are exposed because they are used later to elaborate on some explanation. For example, the SD with the data in the first column (95.22; 95.22; 95.16; 94.52 and 92.88) is 1.00588. The CV value shown in the Table is rounded, the exact CV value is 1.0633; M%IC$_U$ and CV$_U$= for each IC, mean of the percentages identifying the true DGP of the set of c experimental conditions represented in the table, and corresponding CV$_U$. It can be seen that, in this table, M%IC$_U$ and CV$_U$ correspond to the means and CV of S1 in Table 4 of the main text of the article. For the rest, see Table 0.

Table B. Scenario 2. Covariance structure. Percentage of occasions on which the ICs identify the true DGP in [3 CM x 4 MM x 3 SS=36c]. Segregated results from Table 3 of the main text of this article.

	**MM**	**CD**			**MCAR**			**MAR**			**MNAR**				
**CM**	**IC/n_j**	**30**	**45**	**60**	**30**	**45**	**60**	**30**	**45**	**60**	**30**	**45**	**60**	**M%IC$_U$**	**CV$_U$**
**ARH**	**AIC**	69.20	72.50	71.70	67.80	73.10	71.70	69.10	70.80	71.30	69.60	71.30	70.30	70.70	2.19
	**AICC**	75.10	74.60	73.60	72.80	75.80	74	74.90	73.70	71.90	74.40	74.10	72.60	73.96	1.52
	**HQIC**	**90.70**	**92.70**	**93.20**	**90.20**	**93.90**	**93.30**	**92.30**	**93.40**	**97.70**	**90.60**	**91.40**	**93**	92.70	2.16
	**BIC**	**98.90**	**99.10**	**99.40**	**98.30**	**98.90**	**99.30**	**99**	**98.70**	**99.80**	**97.40**	**99**	**99.80**	98.97	.66
	**CAIC**	**100**	**99.90**	**99.70**	**99.70**	**99.80**	**99.80**	**99.80**	**99.90**	**100**	**99.50**	**100**	**100**	99.84	.16
	**Set-M%IC**	86.78	87.76	87.52	85.76	85.76	87.62	87.02	87.30	88.19	86.30	87.16	87.14		
	**Set-CV**	16.12	15.14	15.81	17.12	17.11	15.69	16.28	16.03	17.14	15.72	15.66	16.78		
**TOEPH**	**AIC**	**81.20**	**88.40**	**88.80**	73.50	**85.30**	**86.50**	70.30	**88.40**	**87.30**	70.60	**83.10**	**87.10**	82.54	8,57
	**AICC**	**84.50**	**91.20**	**91.20**	76.80	**88.20**	**89.10**	70.70	**89.10**	**89.70**	72	**85.50**	**88.50**	84.71	8.68
	**HQIC**	**86**	**96.40**	**98.80**	70.20	**89**	**97.10**	60.60	**89**	**94.30**	61.50	**83.70**	**95.40**	85.17	16.01
	**BIC**	66.80	**89.50**	**97.50**	41.50	72	**89**	27.90	69.40	**82.30**	28.30	62.50	**81.60**	67.36	34.94
	**CAIC**	43.10	**80.30**	**93.90**	17.50	52	**81.50**	8.70	28.10	67.70	10.40	31.30	68.80	48.61	61.07
	**Set-M%IC**	72.32	89.16	94.04	55.90	77.30	88.64	47.64	72.80	84.26	48.56	69.22	84.28		
	**Set-CV**	24.91	6.53	4.45	45.90	20.33	6.37	58.62	36.22	12.13	57.04	33.50	11.81		
**UN**	**AIC**	74	**94.98**	**99.30**	73.20	**90.40**	**97.30**	65.10	**85.60**	**94.20**	66.90	**87**	**94.50**	85.21	14.36
	**AICC**	70.40	**94**	**99.20**	67.30	**88.60**	**96.50**	59.70	**82.40**	**93**	59.50	**84.10**	**92.90**	82.30	17.54
	**HQIC**	52.60	**81.90**	**92.60**	46.60	68.30	**85.20**	37	60.70	75.40	38.20	59.60	76.60	64.56	28.60
	**BIC**	23.10	43	66.60	15.90	27.90	47.30	10.30	17.10	33.50	11.40	17.80	35.90	29.15	58.02
	**CAIC**	9.20	21.10	44.10	5	11.60	26.80	2.80	5	15.30	3.40	5.70	15.70	13.81	87.94
	**Set-M%IC**	45.86	67.00	80.36	41.60	57.36	70.62	34.98	50.16	62.28	35.88	50.84	63.12		
	**Set-CV**	62.65	49.60	30.28	72.95	62.55	45.10	80.44	74.19	57.73	78.58	73.73	56.25		

Note. See Tables A.

Table C. Scenario 3. Mean model and covariance structure. Percentage of occasions on which the ICs identify the true DGP in [3 CM x 4 MM x 3 SS=36c]. Segregated results from Table 3 of the main text of this article.

	Mp	CD			MCAR			MAR			MNAR				
**CM**	**IC/n_j**	**30**	**45**	**60**	**30**	**45**	**60**	**30**	**45**	**60**	**30**	**45**	**60**	**M%IC$_U$**	**CV$_U$**
ARH	**AIC**	76.50	77.90	77.80	75.90	76.30	75.90	77.10	77.40	77.40	75.90	75.10	76.10	76.63	1.20
	**AICC**	79.90	79.40	79.30	79.80	77.50	76.80	79.90	79.90	79	79.30	77.40	78.80	78.92	1.38
	**HQIC**	**92.30**	**93.40**	**94.10**	**90.90**	**92.90**	**94.90**	**91.80**	**93.20**	**95**	**91.20**	**93.30**	**94.70**	93.14	1.50
	**BIC**	**99**	**99.10**	**99.40**	**98.50**	**99.10**	**99.50**	**99.10**	**99.30**	**99.50**	**98.20**	**98.90**	**99**	99.05	.39
	**CAIC**	**100**	**99.90**	**99.70**	**99.70**	**99.90**	**99.60**	**99.60**	**99.80**	**99.70**	**99.50**	**99.66**	**99.60**	99.72	.15
	**M%IC**	89.54	89.94	90.10	88.96	89.14	89.34	89.50	89.92	90.12	88.82	88.86	89.64		
	**CV**	12.10	11.81	11.87	12.11	12.91	13.45	11.79	11.84	12.27	12.14	13.27	12.64		
TOEPH	**AIC**	**88**	**92.90**	**93.70**	**80.60**	**91.60**	**92.10**	75.40	**88.30**	**92.20**	75	**88.70**	**93.20**	87.64	7.79
	**AICC**	**89.50**	**94.20**	**94.90**	**80.60**	**92.50**	**93.60**	75.60	**88.80**	**93.60**	75.60	**89.60**	**94.20**	88.56	8.14
	**HQIC**	**88.20**	**96.80**	**98.90**	71.30	**91.80**	**97.30**	61.50	**85.90**	**95.80**	62.90	**86.30**	**95.10**	85.96	15.60
	**BIC**	67	**89.60**	**97.50**	38.20	73.10	**90.20**	26.30	62.70	**81.50**	26	60.70	**83.20**	66.33	37.23
	**CAIC**	43.10	**80.30**	**93.90**	17.10	55.60	**80**	9.60	40.40	67.20	8.80	39.10	70.70	50.48	57.19
	**M%IC**	75.16	90.76	95.78	57.56	80.92	90.64	49.68	73.22	86.06	49.66	72.88	87.28		
	**CV**	26.90	7.05	2.41	49.61	20.20	7.16	60.61	29.12	13.82	61.41	30.68	11.96		
UN	**AIC**	**82.10**	**95**	**99.60**	76.50	**90.50**	**97.50**	66.30	**87**	**94.20**	66	**86.10**	**89.80**	85.88	13.11
	**AICC**	78	**94.20**	**99.50**	70.40	**89.20**	**97.10**	59.80	**83.80**	**93.20**	59.50	**84.10**	**89.80**	83.22	16.45
	**HQIC**	59.10	**81.90**	**92.80**	47.70	70.60	**86.30**	37.70	56.90	74.80	37.50	58.70	69.30	64.44	28.05
	**BIC**	25.60	43	66.70	17.30	28.50	48.80	9.50	19.70	33.30	11.10	19	27.40	29.16	57.26
	**CAIC**	10.40	21.10	44.20	5.10	10	27.40	3.10	5.60	13.40	3.30	5.50	9.80	13.24	92.14
	**M%IC**	51.04	67.04	80.56	43.40	57.76	71.42	35.28	50.60	61.78	35.48	50.68	57.22		
	**CV**	62.38	49.63	30.31	72.78	63.39	44.34	81.04	72.93	59.25	79.00	73.04	64.25		

Note. See Tables A and B.

4 Discussion of the results found in the replicated simulation study

Tables A, B and C exposed in the main text of this research article contain the percentage of occasions on which the ICs identify the mean model, the covariance structure and both the mean model and the covariance structure underlying the true DGP (Scenarios 1, 2, and 3 respectively).

1.-In Scenario 1, where the true DGP underlies an RCL matrix, all the ICs identify the true DGP in more than 90% of the cases, with AIC and AICC being the most efficacious (see Table A). Although all the ICs are sensitive to N and MM in this scenario, the performance is so efficacious that the margin of variation experienced in the worst conditions is very small. These results converge with those found by Gurka (2006) when CM CS, and by Vallejo et al., (2010) when CM is LRC, ARH(1), and UN, in both cases under the full model.

The results contained in the last two rows of Table 1, and also in the last two rows of each Covariance structure in Tables B and C that are presented after, belong to the novel proposal that we present later.

2.-Scenarios 2 and 3 have in common that it is necessary to identify the CM that underlies the data, and in this task, the performance of the ICs is divided. There is a clear difference between the AIC and AICC criteria on the one hand, and BIC and CAIC on the other (see Table B and Table C).

Regarding AIC and AICC, they are the most effective when a TOEPH and UN matrix underlies the true DGP, to practically the same extent. If the N is $n_j \geq 45$ they always identify the true DGP in a percentage greater than 80%. The loss of data affects them systematically when $n_j=30$ and more slightly when the SS is larger. The loss of data affects them systematically when $n_j=30$ and more slightly when the N is larger. However, the same decrease is experienced when the MM is MAR and MNAR, and it is not much greater than when the MM is MCAR. Some of these results converge with those found by Vallejo et al., (2010) and others, with those found by Vallejo et al., (2011).

Concerning CAIC and BIC, as found by Vallejo et al., (2010) when the matrix was AR(1), in ARH(1), these two ICs are the most effective procedures—although the first one is better—in identifying the true DGP in Scenarios 2 and 3. We have verified that this behavior is independent of the SS and the N, in this case.

However, BIC and CAIC, like the case of *Dr. Jekyll and Mr. Hyde* (e.g., CAIC is the most effective IC in ARH(1) and the worst IC in TOEPH and UN), are the ICs most vulnerable to N, MM, and the complexity of underlying CM in the data. If the N is small, the CM underlying the data is complex (TOEPH and UN, it is worse in the latter), and there is loss of data, no matter what the MM, MCAR, MAR, or MNAR, they are useless in identifying the true DGP. However, they are the ICs of choice when an ARH(1) matrix underlies the true DGP since they experience maximum efficacy regardless of the N and MM of the data. Some of these results converge with those found by Vallejo et al., (2010) and others with Vallejo et al., (2011) under the AR(1) and ARH(1) matrix respectively.

3.-The IC HQIC has a behavior that is not like any of the other four ICs. It has a hybrid behavior, in the following sense. When the matrix is ARH(1), it experiences a performance similar to that of BIC and CAIC, but it identifies the true DGP on fewer

occasions. When the matrix is TOEPH and UN, its behavior is close to that performed by AIC and AICC, but it is more vulnerable than these two to N and MM, especially when the matrix is UN. Therefore, it is the only one of the five ICs that could be the IC of choice in any of the situations manipulated here as long as $n_j \geq 45$ regardless of the MM, except when a UN matrix underlies the true DGP, and the loss of data occurs under MAR or MNAR mechanisms. This result also converges with the benchmark investigations.

4.- One aspect that seems very relevant to us is that in all the investigations referred to, the importance of the N has been highlighted, and in this case too. Vallejo et al., (2010) and Vallejo et al., (2011) also highlighted that the performance of the ICs was better the higher the number of repeated measures. In this case, we have verified that n_j =45 is sufficient for the ICs to reach a reasonable efficacy where each IC performs at its best, and that better results are achieved with t=5 than with t=4, however, the efficacy is not much lower than with t=8 or 12 (in both cases, using the referenced research as a benchmark). Both values (n_j =45 and t=5) may be sufficient to be able to portray the CM structure that underlies the data. Vallejo et al., (2010) concluded that the optimal N would be (t x 10). In this case, it was sufficient.

5 References

Gurka, M.J., 2006. Selecting the Best Linear Mixed Model Under REML. *The American Statistician*, 60(1), pp.19–26. Available at: https://dx.doi.org/10.1198/000313006x90396.

Livacic-Rojas, P., Fernández, P., Vallejo, G., Tuero-Herrero, E. and Ordóñez, F., 2020. Sensitivity of five information criteria to discriminate covariance structures with missing data in repeated measures designs. *Psicothema*, 32(3), pp.399–409. Available at: https://doi.org/10.7334/psicothema2020.63.

Vallejo, G., Arnau, J., Bono, R., Fernández, P. and Tuero-Herrero, E., 2010. Nested model selection for longitudinal data using information criteria and the conditional adjustment strategy. *Psicothema*, 22(2), pp.323–333.

Vallejo, G., Fernández, M.P., Livacic-Rojas, P.E. and Tuero-Herrero, E., 2011. Selecting the best unbalanced repeated measures model. *Behavior Research Methods*, 43(1), pp.18–36. Available at: https://dx.doi.org/10.3758/s13428-010-0040-1.
